# RNA editing-induced structural and functional adaptations of NAD9 in *Triticum aestivum* under drought stress

**DOI:** 10.3389/fpls.2024.1490288

**Published:** 2024-11-06

**Authors:** Nermin G. Mohamed, Ahmed M. Ramadan, Marwa Amer, Yasser Morsy, Rasha A. Mohamed, Osama A. M. Said, Afnan A. Alnufaei, Mona I M. Ibrahim, Sameh E. Hassanein, Hala F. Eissa

**Affiliations:** ^1^ College of Biotechnology, Misr University for Science and Technology (MUST), Giza, Egypt; ^2^ Biological Science Department, Faculty of Science, King Abdulaziz University, Jeddah, Saudi Arabia; ^3^ Princess Najla bint Saud Al-Saud Center for Excellence Research in Biotechnology, King Abdulaziz University, Jeddah, Saudi Arabia; ^4^ Department of Gastroenterology and Hepatology, University Hospital Zurich, University of Zurich, Zurich, Switzerland; ^5^ Agricultural Genetic Engineering Research Institute (AGERI), Agricultural Research Center (ARC), Giza, Egypt; ^6^ Department of Biology, Faculty of Science, University of Bisha, Bisha, Saudi Arabia; ^7^ Bioinformatics Program, School of Biotechnology, Nile University, Giza, Egypt

**Keywords:** RNA editing, *nad9* gene, drought stress, *Triticum aestivum*, post-translational modification

## Abstract

**Introduction:**

Mitochondria are essential organelles in eukaryotic cells, producing ATP through the electron transport chain to supply energy for cellular activities. Beyond energy production, mitochondria play crucial roles in cellular signaling, stress responses, and the regulation of reactive oxygen species. In plants, mitochondria are one of the keys to responding to environmental stresses which can significantly affect crop productivity, particularly in crops like wheat. RNA editing, a post-transcriptional RNA modification process in mitochondria, is linked to regulating these stress responses.

**Methods:**

This study explores RNA editing patterns in the *nad9* gene of wheat drought-tolerant (Giza168) and drought-sensitive (Gemmiza10) wheat cultivars under drought stress to understand plant adaptation mechanisms. RNA-seq data for these cultivars were analyzed using CLC Genomic Workbench to identify RNA editing sites in the *nad9* gene, examining subsequent amino acid changes and predicting secondary structure modifications. These RNA editing sites were validated using qRT-PCR on drought-treated seedlings at 0, 2, and 12 hours post-treatment. Protein models were generated using AlphaFold, with functional predictions and structure verification conducted using various bioinformatics tools to investigate the effect of RNA editing on protein level.

**Results:**

The results showed significant RNA editing events, especially C-to-T conversions, in the *nad9* gene across different drought exposure times. Giza168 had 22 editing sites, while Gemmiza10 had 19, with several showing significant differences between control and stress conditions. RNA editing influenced the NAD9 protein's secondary structure, particularly beta sheets, and 3D modeling highlighted the structural impacts of these edits. The N-terminal region of NAD9 contained important regulatory motifs, suggesting a complex regulatory environment.

**Discussion:**

This study reveals key editing sites that differ between drought-tolerant and sensitive wheat cultivars, impacting NAD9 protein structures and highlighting the role of RNA editing in enhancing drought resilience. Additionally, the study suggests potential regulatory mechanisms, including phosphorylation and ubiquitination that influence mitochondrial stability and function.

## 1 Introduction

Mitochondria play a critical role in eukaryotes by hosting some of the most vital biochemical pathways essential for cellular function. These organelles are central to the production of adenosine triphosphate (ATP) through the electron transport chain, which serves as the primary energy source for the cell. Beyond energy production, mitochondria are involved in cellular signaling, stress responses, reactive oxygen species (ROS) regulation, biosynthesis of essential biomolecules, photorespiration, and programmed cell death (Qin et al., 2021; Rasool et al., 2022). Mitochondrial functions are also critical targets for abiotic stresses, which are major environmental factors that limit plant growth and productivity (Munns and Millar, 2023). Furthermore, mitochondria are crucial in plant cell adaptation to abiotic stresses, such as drought and salinity, which induce oxidative stress at the cellular level (Xiong et al., 2017). Energy-dissipating mechanisms regulate the production of ROS in plant mitochondria, and respiration plays a pivotal role in synthesizing ascorbate, an antioxidant critical for detoxifying ROS (Rasool et al., 2022).

Global climate change exacerbates various abiotic stress conditions, including droughts, floods, and salinity (Munns and Millar, 2023). Drought is one of the most detrimental environmental stresses, significantly limiting plant growth and threatening global agricultural production by causing substantial yield losses in major crops such as wheat (Trenberth et al., 2014). Wheat, a staple crop consumed by nearly half of the world’s population, is particularly vulnerable to drought stress, severely impacting its productivity (Rasool et al., 2022). Various biotic and abiotic stress factors, including drought, challenge wheat and other crops, affecting their agronomic performance and productivity (Xiong et al., 2017; Pandey et al., 2017).

In higher plants, RNA editing is a post-transcriptional modification primarily occurring in mitochondrial- and chloroplast-encoded transcripts, where cytidine residues are converted into uridine. This editing process ensures the homology of encoded proteins and is crucial for gene expression (Qin et al., 2021). RNA editing is observed in coding sequences and untranslated regions, tRNAs, and rRNAs, with significant implications for protein function and cellular stress responses (Edera et al., 2018; Xiong et al., 2017). RNA editing in plant mitochondria is linked to regulating environmental stress responses, including oxidative stress caused by abiotic factors such as drought and salinity (Rasool et al., 2022).

The phosphorylating NADH dehydrogenase (Complex I) and non-phosphorylating NAD(P)H dehydrogenases are vital components of the NAD(P)H oxidation pathways in plant mitochondria (Xiong et al., 2017). The NAD protein complex plays a crucial role in cellular respiration, particularly in the oxidative phosphorylation pathway, which involves a series of redox reactions that generate the proton gradient necessary for ATP synthesis (Ghifari and Murcha, 2020). These reactions occur primarily in the inner mitochondrial membrane, facilitated by large protein complexes within the electron transport chain (Ramadan, 2020).

Research has shown that RNA editing of NADH dehydrogenase subunits, such as NAD9, occurs in plant mitochondria and is associated with varying stress responses. For example (Xiong et al., 2017), identified 90 RNA editing sites in *nad9* transcripts in six mitochondrial genes across two rice varieties, many of which were stress-responsive. Such findings underscore the importance of RNA editing in mitochondrial function and stress adaptation, particularly under drought conditions (Rasool et al., 2022).

The present study aims to investigate *nad9* RNA editing patterns in wheat in response to drought stress and compare these patterns between drought-tolerant and sensitive wheat cultivars. This research aims to enhance our understanding of how RNA editing contributes to plant adaptation to environmental stresses, potentially guiding the development of more resilient crop varieties.

## 2 Materials and methods

### 2.1 RNA-seq data acquisition

RNA-seq data for *Triticum aestivum* were retrieved from the Sequence Read Archive (SRA) databank available through the National Center for Biotechnology Information (NCBI). The specific datasets included SRR3089142 and SRR3089143 (G168 control), SRR3089150 and SRR3089151 (GM10 control), SRR3089146 and SRR3089147 (G168 after 2 hours of drought stress), SRR3089152 and SRR3089153 (GM10 after 2 hours of drought stress), SRR3089148 and SRR3089149 (G168 after 12 hours of drought stress), and SRR3089144 and SRR3089145 (GM10 after 12 hours of drought stress).

### 2.2 In silico RNA editing analysis

RNA editing sites were identified using CLC Genomic Workbench version 21.0.3 (Qiagen, Denmark). The mapping parameters were set with a similarity threshold of 98% and a length fraction of 98% to exclude undesired reads. The reads were aligned to the *Triticum aestivum* G168 mitochondrial *nad9* gene (GenBank accession no. OQ079951) and GM10 mitochondrial *nad9* gene (GenBank accession no. OQ079955) as described by Hisano et al. (2016). The minimum similarity fraction was set at 80%, and the minimum read length fraction at 50%. Nucleotide editing parameters were defined as follows: 5% low-frequency variant, 20% minimum coverage, 4% minimum count, and 5% minimum frequency. The RNA editing sites, total read counts, and coverage depth counts were subsequently established. The nucleotide conversion frequency at each editing site was calculated across all drought exposure periods and compared to the control using the ratio of nucleotide conversion reads to total reads (Wang et al., 2015).

### 2.3 Analysis of nad9 RNA editing-derived amino acid changes

Genomic and complementary DNA (cDNA) sequences of the *nad9* gene were analyzed using CLC Genomic Workbench 21.0.3 to identify RNA editing sites and resultant amino acid changes. The same software also predicted secondary structure modifications in proteins.

### 2.4 RNA editing site validation via qRT-PCR

To validate RNA-seq predicted editing sites, drought stress was applied to nine-day-old seedlings of the drought-tolerant wheat cultivar Giza168 and the moderately drought-tolerant cultivar Gemmiza10. The seedlings were treated with Hoagland solution containing 20% w/v polyethylene glycol (PEG-6000) to simulate drought conditions. Leaf samples from individual plants were collected in triplicate at 0, 2, and 12 hours post-treatment. Tissues were flash-frozen in liquid nitrogen and stored at -80°C until further analysis. Total RNA was extracted using Qiazol (Qiagen, Cat No. 79306), and RNA editing sites were validated using the Mx3005P qPCR system (Stratagene). First-strand cDNA synthesis was performed using 1 µg of total RNA, 0.5 µg of reverse primers for each gene (**Supplementary Table S1**), and M-MuLV reverse transcriptase (MIR BIOTECH, Cat. No. chb20004) as per the method of Slugina et al. (2019). Amplification was conducted in 25 µL reaction volumes, containing 12.5 µL RT2 SYBR-Green qPCR Mastermix, 0.2 µM of each gene-specific primer, and PCR-grade water to a total volume of 24 µL, with 1 µL of diluted cDNA as the template. The qRT-PCR cycling conditions were 94°C for 15 s, 55°C for 30 s, and 72°C for 45 s over 40 cycles. Data analysis was performed by generating amplification plots of ΔRn versus cycle number, and RNA editing percentages were calculated as described by (Rodrigues et al., 2017).


$$\%\text{RNA}\;\text{editing}\;=\left\{2^{\left(\text{Ct}\;\text{mean}\;\text{of}\;\text{T}\;\text{variant}\;\text{‐}\;\text{Ct}\;\text{mean}\;\text{of}\;\text{C}\;\text{variant}\right)}/2^{\left(\text{Ct}\;\text{mean}\;\text{of}\;\text{T}\;\text{variant}\;\text{‐}\;\text{Ct}\;\text{mean}\;\text{of}\;\text{C}\;\text{variant}\right)}+1\right\}\;100$$

### 2.5 3D modeling and functional prediction

Protein models for all treatments were generated using AlphaFold, a state-of-the-art deep-learning approach for accurate protein structure prediction (Jumper et al., 2021). Functional predictions of the unstructured N-terminus loop were conducted using the ELM (Eukaryotic Linear Motif) resource (Gould et al., 2010). The protein structures were visualized using PyMOL (Alexander et al., 2011), and model verification was conducted with MolProbity (Chen et al., 2010). Align Uniprot (2023) and PROMALS3D (Pei et al., 2008) were utilized for sequence alignment.

### 2.6 Statistical analysis

Data were statistically analyzed using SPSS software, with analysis of variance (ANOVA) followed by Tukey’s Honestly Significant Difference (HSD) test for *post-hoc* comparisons (Tukey, 1949).

## 3 Results

### 3.1 Detection of *nad9* transcripts in wheat

Genomic and cDNA sequences of the *nad9* gene were successfully recovered for the Giza168 cultivar, which is known for its drought tolerance, under different conditions: control (GenBank acc. no. OQ079952), 2 hours after drought stress (GenBank acc. no. OQ079953), and 12 hours after drought stress (GenBank acc. no. OQ079954). The RNA sequencing data consisted of approximately 170,200,000 paired-end reads for the control condition, 170,450,000 for the 2-hour drought stress condition, and 172,700,000 for the 12-hour drought stress condition.

For the Gemmiza10 cultivar, which is more susceptible to drought, genomic and cDNA sequences of the *nad9* gene were also retrieved under similar conditions: control (GenBank acc. no. OQ079956), 2 hours after drought stress (GenBank acc. no. OQ079957), and 12 hours after drought stress (GenBank acc. no. OQ079958). The corresponding RNA sequencing data included 172,880,000 paired-end reads for the control, 173,800,000 for the 2-hour stress condition, and 172,900,000 for the 12-hour stress condition.

### 3.2 RNA editing, validation and amino acid modifications

**Figure 1:**
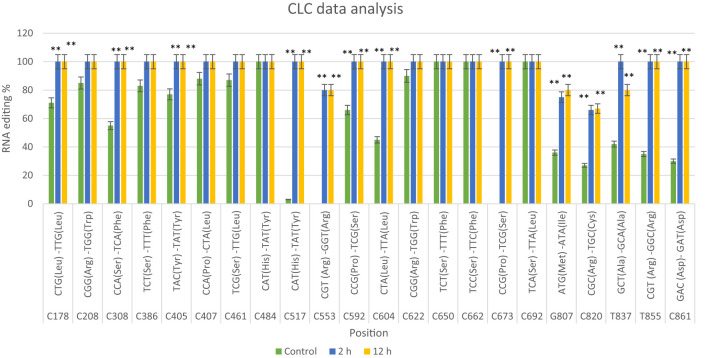
CLC data analysis of the *nad9* gene of *Triticum aestivum* cultivar Giza168 editing sites at three different times of drought (control, 2 hours after drought exposure, 12 hours after drought exposure). Data are expressed as means with ± SD (black bars) of three biological replicates. ** indicate significant difference between treatments (P < 0.01).

RNA editing events were analyzed by comparing the mitochondrial *nad9* DNA sequence with its cDNA sequence after 2 and 12 hours of drought exposure in the Giza168 cultivar. The analysis revealed 22 editing sites, including 17 C-to-T conversions (e.g., at positions C178, C208, C308), and additional conversions such as G-to-C, C-to-G, G-to-A, T-to-A, and T-to-C at specific positions. Of these, 16 editing sites showed significant changes after 2 and 12 hours of drought treatment, whereas six sites exhibited non-significant differences. Editing ratios varied with drought exposure and duration (**Figure 1**; **Supplementary Figure S1**; **Supplementary Tables S2**, **S4**).

**Figure 2:**
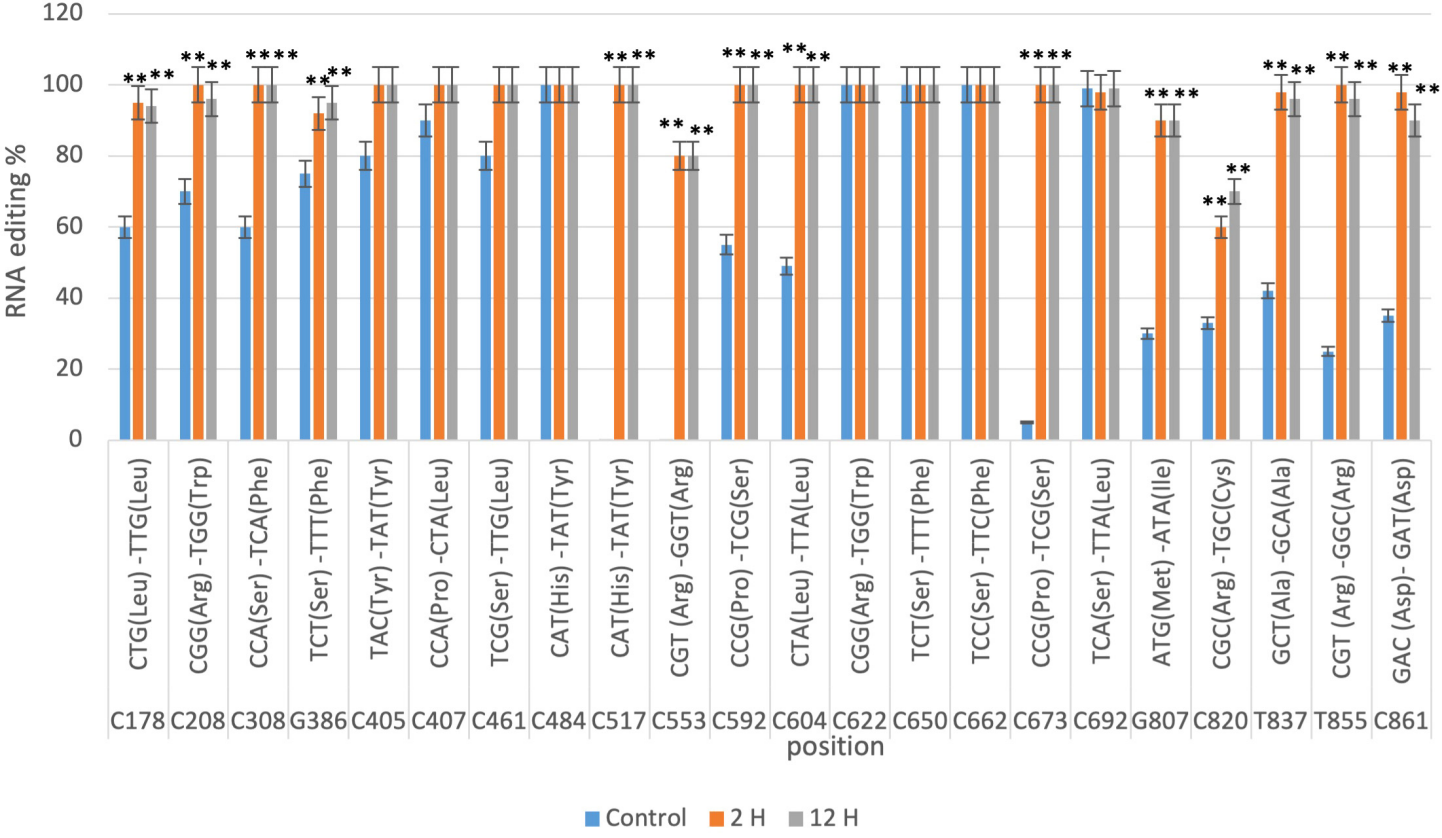
qRT-PCR confirmation of *nad9* RNA editing sites in the *Triticum aestivum* cultivar Giza168 predicted by CLC genomic workbench in three different times of drought (control, 2 hours after drought exposure, 12 hours after drought exposure). Data are expressed as means with ± SD (black bars) of three biological replicates. ** indicate a significant difference between treatments (P < 0.01).

The RNA editing sites identified in the *nad9* gene were validated using qRT-PCR for Giza168 cultivar. The editing positions were quantified and analyzed at three-time intervals (control, 2 hours, and 12 hours), with the data confirming the RNA-seq predictions. In Giza168 (**Figure 2**), the analysis revealed a significant increase in RNA editing at several key positions after drought exposure, particularly at the 2-hour and 12-hour marks. For instance, sites C178, C208, and C308 showed near-complete editing after 12 hours of drought treatment, with editing rates exceeding 90%, compared to lower editing percentages in the control condition. Similarly, sites such as C517 and C553 exhibited significant changes in editing percentages (P < 0.01) across all time points, highlighting the role of RNA editing in regulating gene expression in response to drought stress.

**Figure 3:**
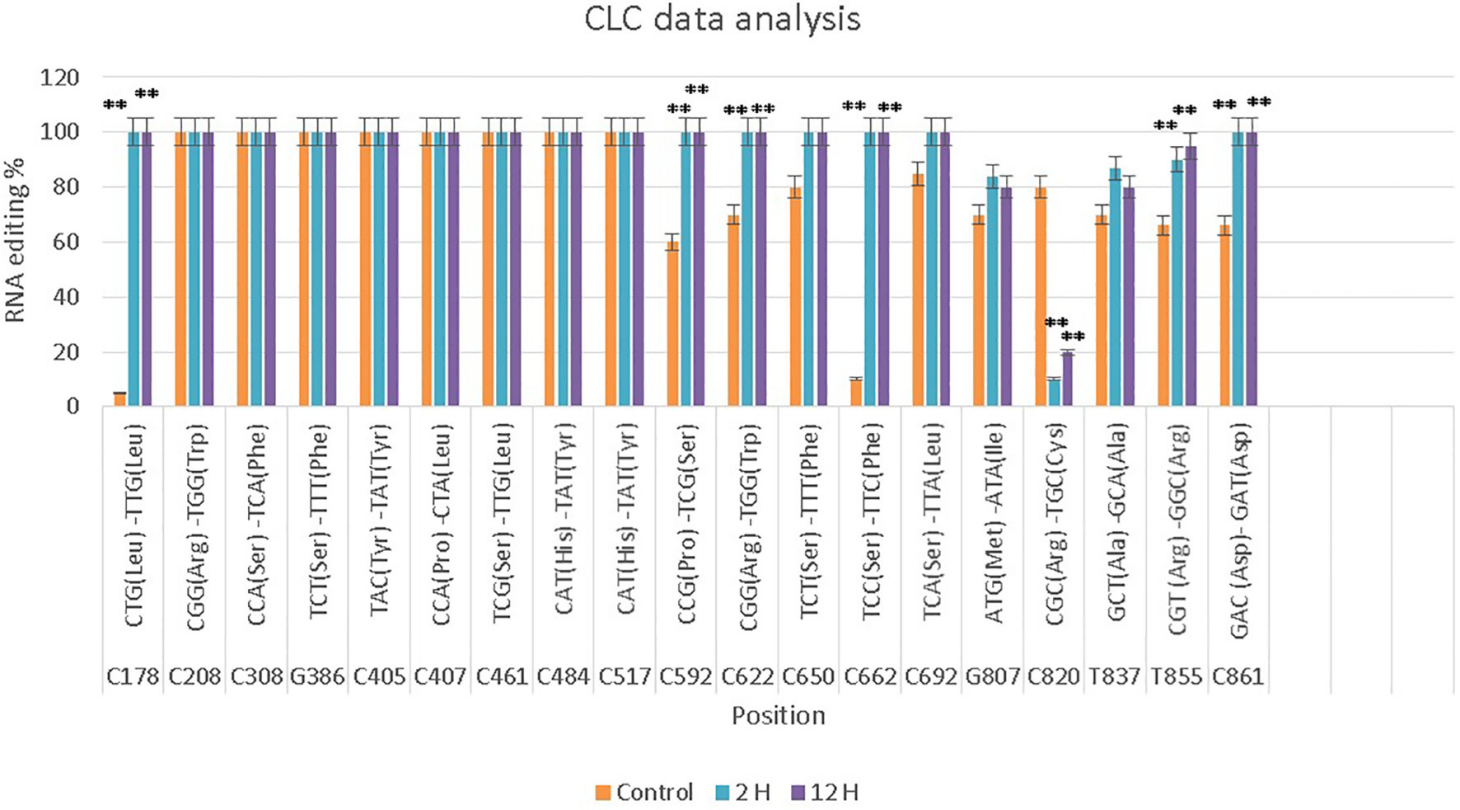
CLC data analysis of the *nad9* gene of *Triticum aestivum* cultivar Gemmiza10 editing sites at three different times of drought (control, 2 hours after drought exposure, 12 hours after drought exposure). Data are expressed as means with ± SD (black bars) of three biological replicates. ** indicate a significant difference between treatments (P < 0.01).

In the Gemmiza10 cultivar, 19 RNA editing sites were identified, with 15 C-to-T conversions and several other nucleotide changes. Eight sites exhibited significant differences across the time points, while the rest showed non-significant differences. Editing at specific codons, such as C178 and C405, was consistently observed across both cultivars (**Figure 3**; **Supplementary Figure S2**; **Supplementary Tables S3**, **S4**).

**Figure 4:**
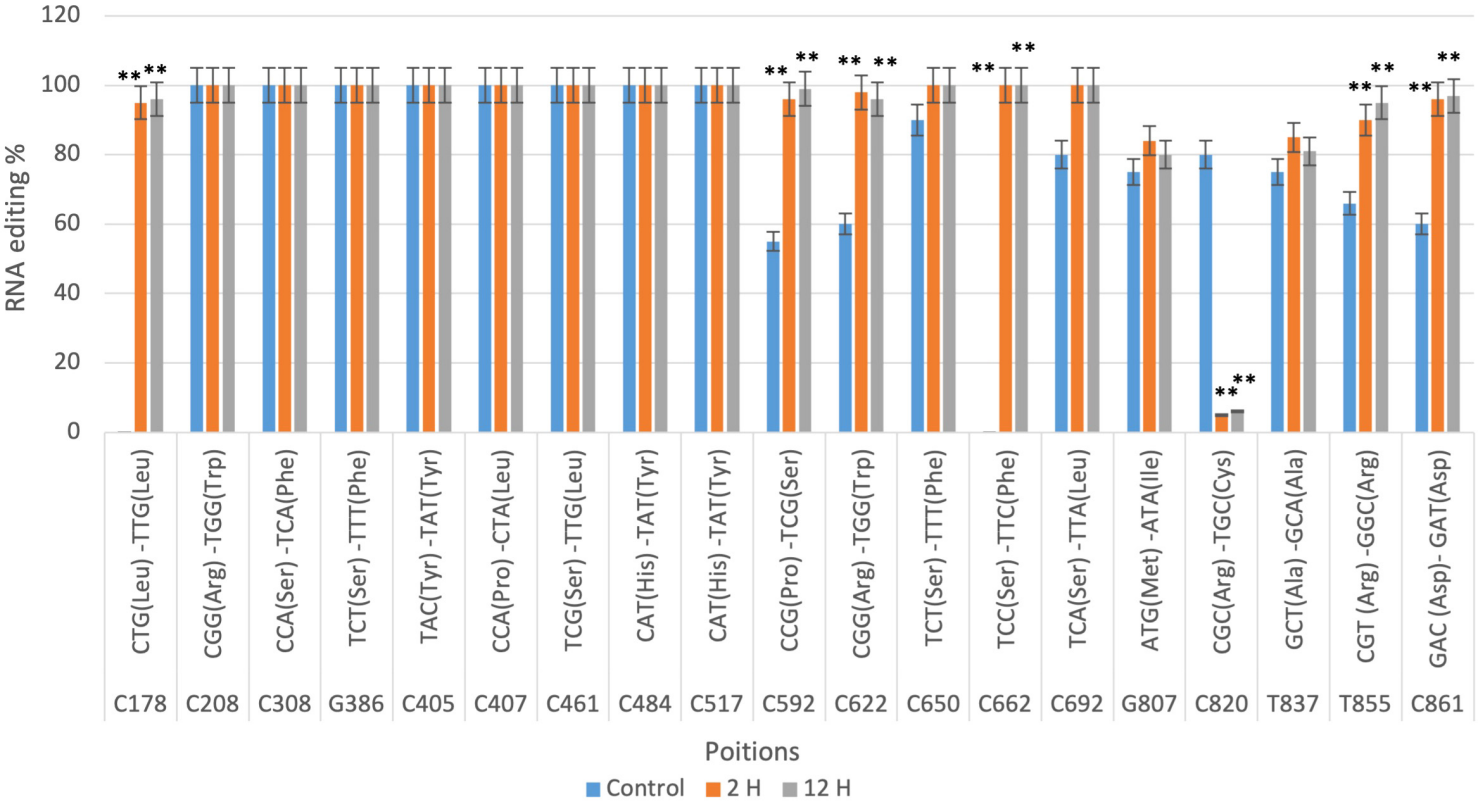
qRT-PCR confirmation of *nad9* RNA editing sites in the Triticum aestivum cultivar Gemmiza10 predicted by CLC genomic workbench in three different times of drought (control, 2 hours after drought exposure, 12 hours after drought exposure). Data are expressed as means with ± SD (black bars) of three biological replicates. ** indicate a significant difference between treatments P < 0.01.

In contrast, the RNA editing sites in drought-sensitive cultivar Gemmiza10 is validated by qRT-PCR (**Figure 4**). While RNA editing at positions such as C178 and C208 also reached high levels in the 12-hour treatment, several sites, including C820 and C850, exhibited lower editing percentages under drought conditions compared to Giza168. This discrepancy between cultivars suggests that RNA editing might contribute to the differential drought responses observed in these two wheat varieties. Significant differences between treatments were confirmed by qRT-PCR, as indicated by the statistical markers (**P < 0.01) in **Figure 4**.

### 3.3 secondary structure of the NAD9 protein

**Figure 5:**
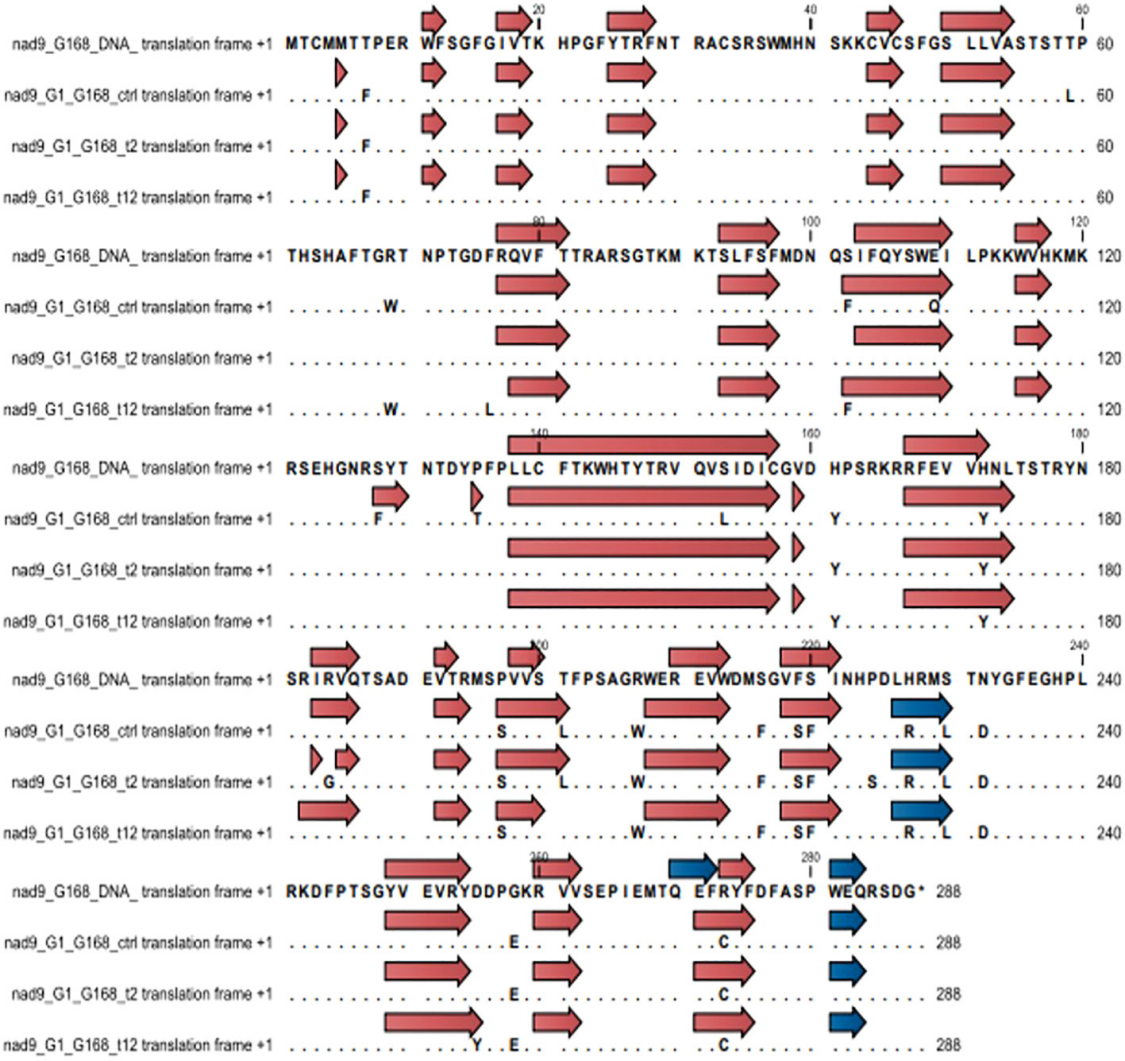
Changes in secondary structure regions in cultivar Giza168 (G168) NAD subunit 9 before and after RNA editing due to drought treatment. Blue and brown arrow heads indicate alpha helices and beta sheets, respectively.

**Figure 6:**
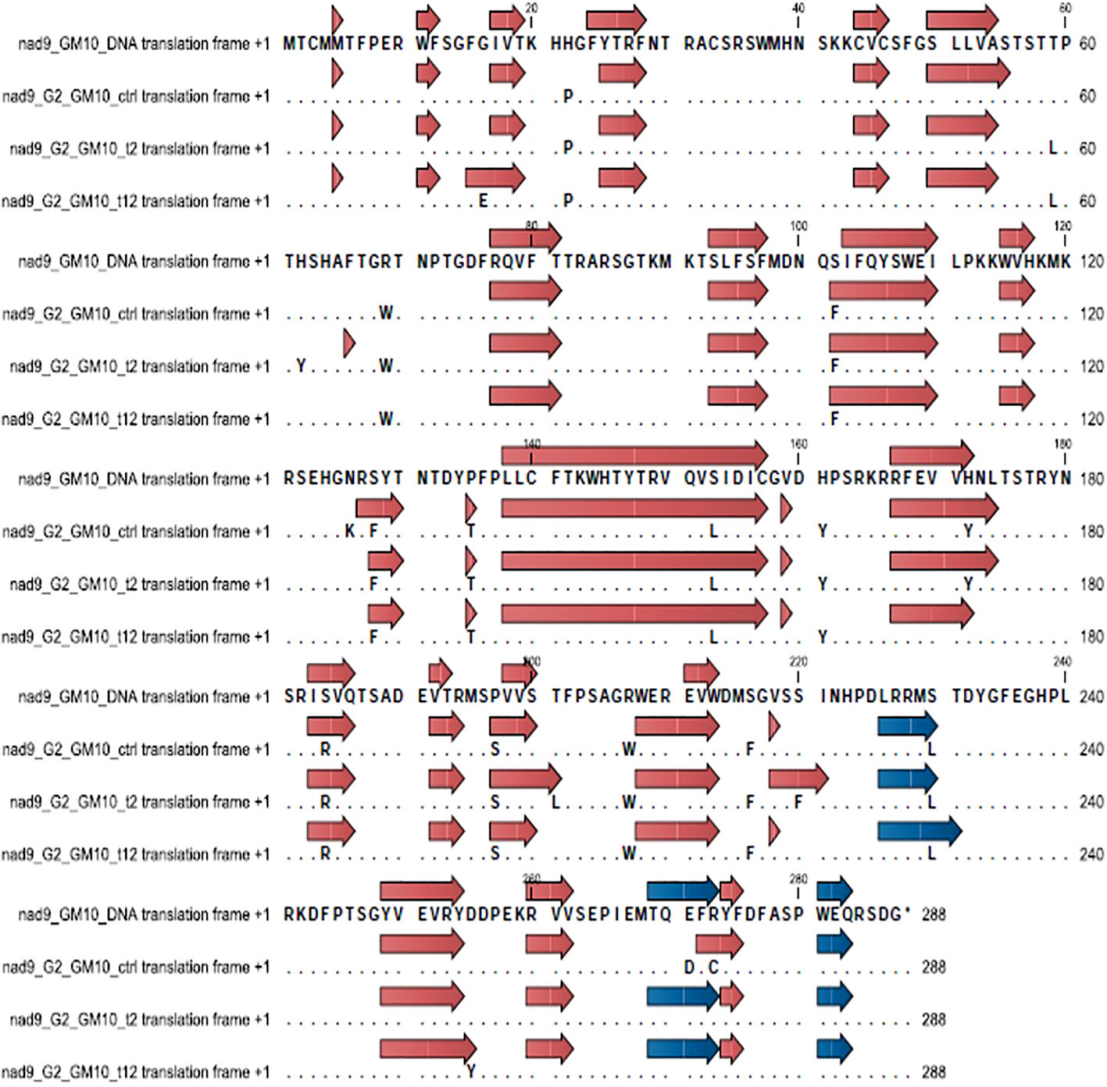
Changes in secondary structure regions in cultivar Gemmiza10 (GM10) NAD subunit 9 before and after RNA editing to drought treatment. Blue and brown arrow heads indicate alpha helices and beta sheets, respectively.

Secondary structural changes in the NAD9 protein due to RNA editing were analyzed using CLC Genomic Workbench. In the Giza168 cultivar, modifications in beta sheets were observed under different drought conditions, with a decrease in the number of beta sheets from 23 in the control to 21 after 12 hours of drought. Notably, specific beta sheets, such as those at positions 228-230, were only present in the control condition. No significant changes were observed in the number of alpha helices, although some shifts in their locations were detected. Similar structural modifications were observed in the Gemmiza10 cultivar, with changes in the number and position of beta sheets and alpha helices under drought conditions (**Figures 5**, **6**; **Supplementary Figures S3**, **S4**).

### 3.4 3D structure of the NAD9 protein

#### 3.4.1 NAD9 protein modeling

**Figure 7:**
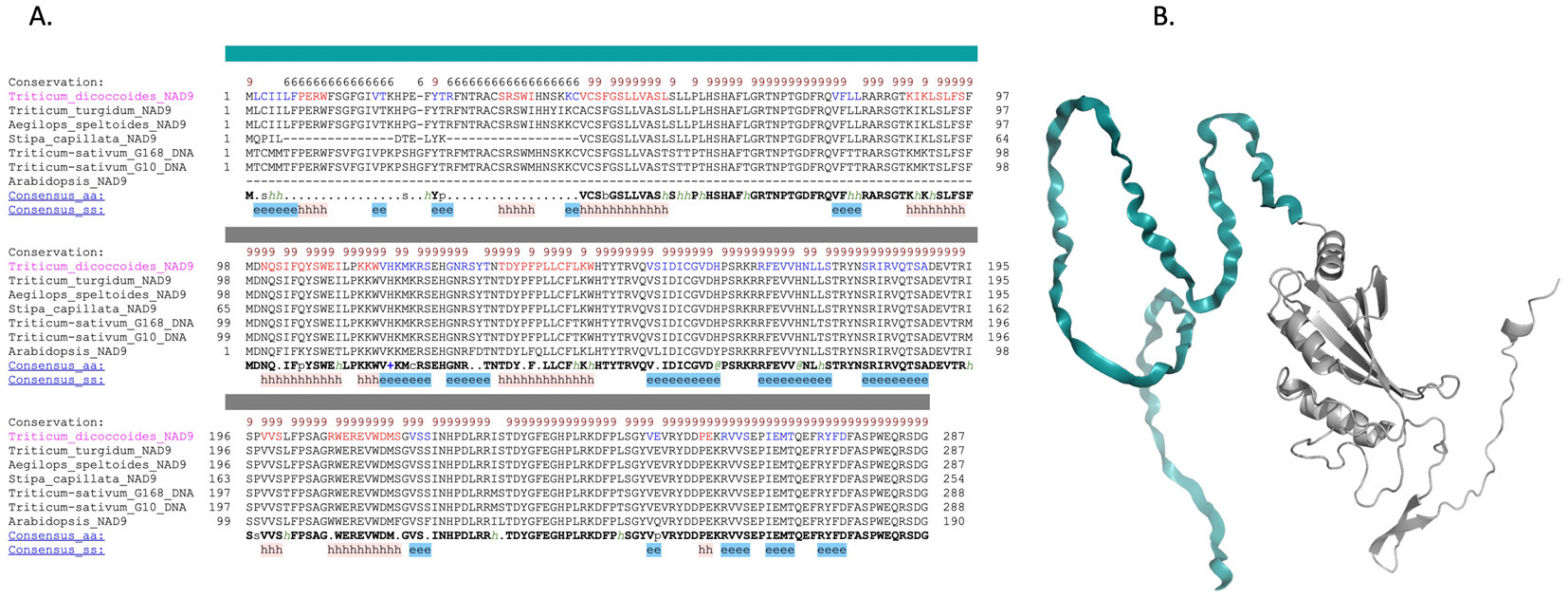
Comparative analysis and structural modeling of the NAD9 protein across species. **(A)** Multiple sequence alignment of the NAD9 protein from seven diverse species, highlighting a 98-amino acid (in dark color) region unique to *Triticum aestivum*. The alignment is annotated to indicate conserved alpha helices (represented by ‘h’) and beta strands (‘e’), with conserved residues highlighted against the consensus sequence at the bottom. Complete conservation across species is denoted by an asterisk above the alignment. **(B)** Ribbon diagram depicting the NAD9 protein structure in *Triticum aestivum*, detailing the arrangement of alpha helices and beta strands corresponding to the annotated sequence alignment.

Due to the inability of AlphaFold to accurately model the N-terminal region of the NAD9 protein, multiple sequence alignment (MSA) was used to discern the structural significance of the protein (**Figure 7**). These MSAs juxtapose sequences from various plant species, including *Arabidopsis*, *Stipa capillata*, *Triticum turgidum*, *Triticum dicocoides*, and *Aegilops*. Notably, the *Arabidopsis* structure did not encompass the initial 98 amino acids in *Triticum aestivum* Gemmiza10 or *Triticum aestivum* Giza168. This model provides a visual reference for the protein’s secondary structure, with the species-specific structural attributes indicating how sequence variations manifest in the protein’s three-dimensional conformation. MSA inferred the potential secondary structure of these 98 amino acids, aligning with prior data (**Figures 5**, **6**). This inferred secondary structure provides insight into the likely configuration of the N-terminal region, filling the gaps left by the incomplete structural model.

#### 3.4.2 N-terminal NAD9 in *Triticum aestivum*: implications for mitochondrial function

**Figure 8:**
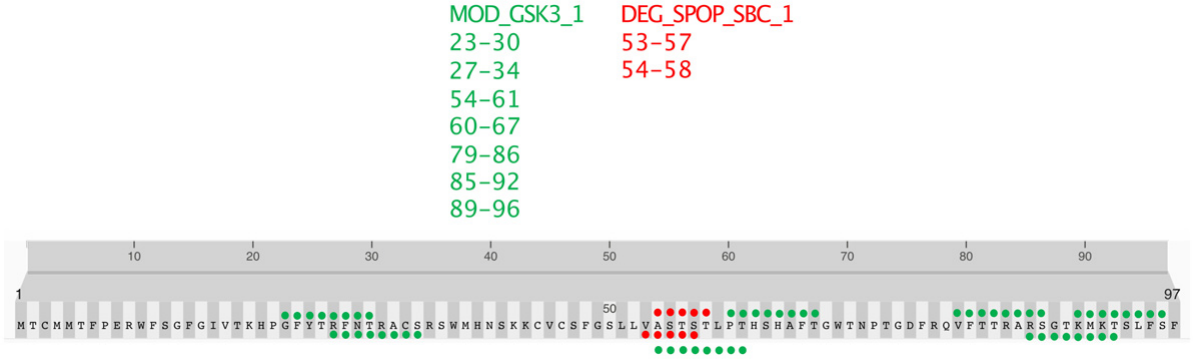
Predicted post-translational modification motifs in the N-terminal region of NAD9. The localization of predicted post-translational modification motifs within the first 98 amino acids of the NAD9 protein from *Triticum aestivum*. GSK3 phosphorylation sites (MOD_GSK3_1) are marked in green, and SPOP-binding consensus motifs (DEG_SPOP_SBC_1) are indicated in red. The positions of each motif are numerically labeled. The amino acids 1-98 shown in this figure is directly based on the nad9 DNA sequence without considering RNA editing.

This study’s detailed bioinformatic analysis of the N-terminal peptide (amino acids 1-98) of the NAD9 protein from *Triticum aestivum* revealed a constellation of posttranslational modification sites, which may confer intricate regulatory capabilities. Notably, a serine/threonine-rich motif corresponding to the SPOP-binding consensus (SBC) was identified (**Figure 8**). This motif, typically embedded within regions of intrinsic disorder in substrate proteins, suggests potential regulation via the SPOP/Cul3-dependent ubiquitination pathway.

The analysis further highlighted two predominant types of motifs: MOD_GSK3_1, indicative of potential GSK3 kinase phosphorylation sites, depicted in green, and DEG_SPOP_SBC_1, suggestive of potential ubiquitination sites via the SPOP/Cul3 pathway (**Figure 8**). These motifs are located at positions 53–57 and 54–58 and are highlighted in red.

Moreover, the analysis of the N-terminus of the NAD9 protein from *Triticum aestivum* revealed several predicted GSK3 phosphorylation sites. The predicted phosphorylation sites by GSK3 (MOD_GSK3_1) are distributed across the sequence at the following positions: 23–30, 27–34, 54–61, 60–67, 79–86, 85–92, and 89–96 (**Figure 8**). These predicted sites are highlighted in green on the protein sequence. The distribution of GSK3 phosphorylation sites throughout the N-terminal sequence strongly regulates phosphorylation, which could significantly impact the protein’s role and interactivity within the mitochondrial complex. In contrast, the SPOP-binding consensus motifs are nearby, though fewer, forming a cluster that may serve as a focal point for regulatory control, mediating protein stability and signaling cascades within the cell.

#### 3.4.3 RNA editing and its impact on the NAD9 protein structure in the *Triticum aestivum* Giza168 cultivar

**Figure 9:**
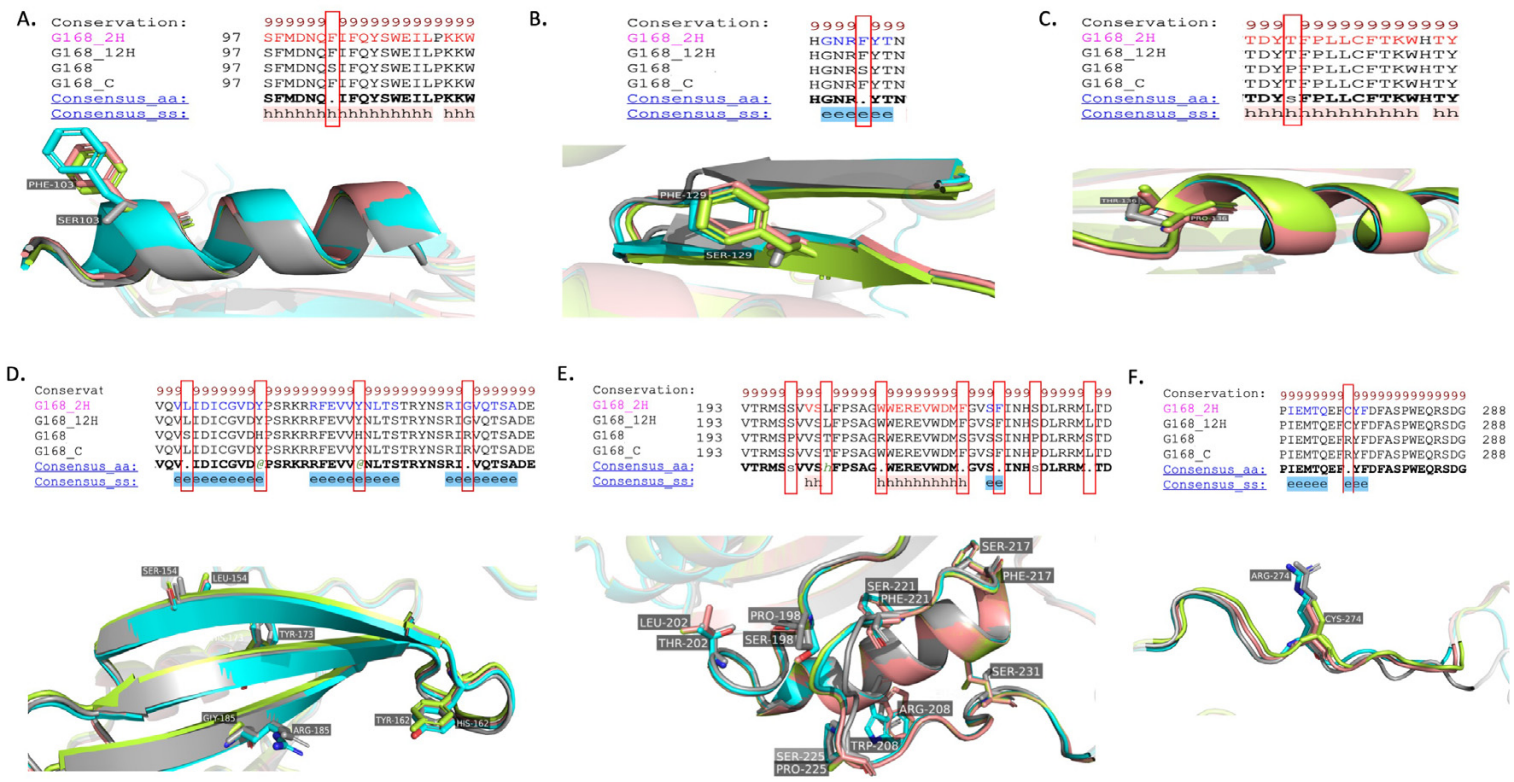
Structural analysis of RNA editing variants in the NAD9 protein of the *Triticum aestivum* Giza168 cultivar across the three drought treatment time points (control, 2 hours, 12 hours). **(A-F)** Structural variants resulting from RNA editing in the NAD9 protein of the *Triticum aestivum* Giza168 (G168) cultivar were analyzed through alignment with the NAD9 protein virtual translated from DNA (G168_ gray) and three additional models: NAD9_G168_control (G168_C) in cyan, NAD9_G168_2H (G168_2H) in the Limon, and NAD9_G168_12H (G168_12H) in the dark salmon. **(A)** Detailed view of an alpha-helix region showing conserved RNA editing sites across different treatment time points (control, 2 hours, 12 hours). **(B)** Ribbon diagrams of the protein structure around RNA editing sites with accentuated changed residues, illustrate the potential impact on the local structure. **(C)** no-effect in alpha helix integrity due to RNA editing. **(D)** Beta-strand representation of RNA editing sites showing the conservation of structural changes. **(E, F)** C-terminal region alignment, emphasizing the conserved RNA editing sites and their structural significance.

Detailed observations of RNA editing within the NAD9 protein of the *Triticum aestivum* Giza168 cultivar have been observed, highlighting specific amino acid changes and their respective positions within the protein sequence (**Figure 9**). In Panel A, an edit at position 103 changes a serine (S) in the DNA sequence to a phenylalanine (F) in the protein. This may influence the hydrophobicity and protein interaction at this site within the alpha-helical region. Panel B illustrates an editing event at position 129, where serine (S) is also converted to phenylalanine (F), suggesting a possible conserved editing mechanism to alter the dynamic properties of the alpha helix. In Panel C, proline (P) at position 136 is changed to threonine (T) a change that could impact the local structure by introducing a polar side chain. Panel D presents multiple editing sites: at position 154, serine (S) is changed to leucine (L); at position 162, histidine (H) is replaced by tyrosine (Y); at position 173, a histidine (H) change tyrosine (Y)” is observed; and at position 185, arginine (R) is altered to glycine (G) at the 2-h and 12-h time points, which may affect beta-strand stability and intermolecular interactions. In panel E, the substitutions at residue 198, which changes proline (P) to serine (S); at residue 202, which changes threonine (T) to leucine (L); at residue 208, which changes arginine (R) to tryptophan (W); at residues 217 and 221, which changes serine (S) to phenylalanine (F); and at residue 225, which changes proline (P) to serine (S), are displayed for the 2 hours and 12 hours drought treatments. At position 231, serine (S) is also changed to leucine (L) Panel F shows a change at position 274 from arginine (R) to cysteine (C) in the 2-hour and 12-hour drought treatments.

#### 3.4.4 RNA editing and its impact on the NAD9 protein structure in the *Triticum aestivum* Gemmiza10 cultivar

**Figure 10:**
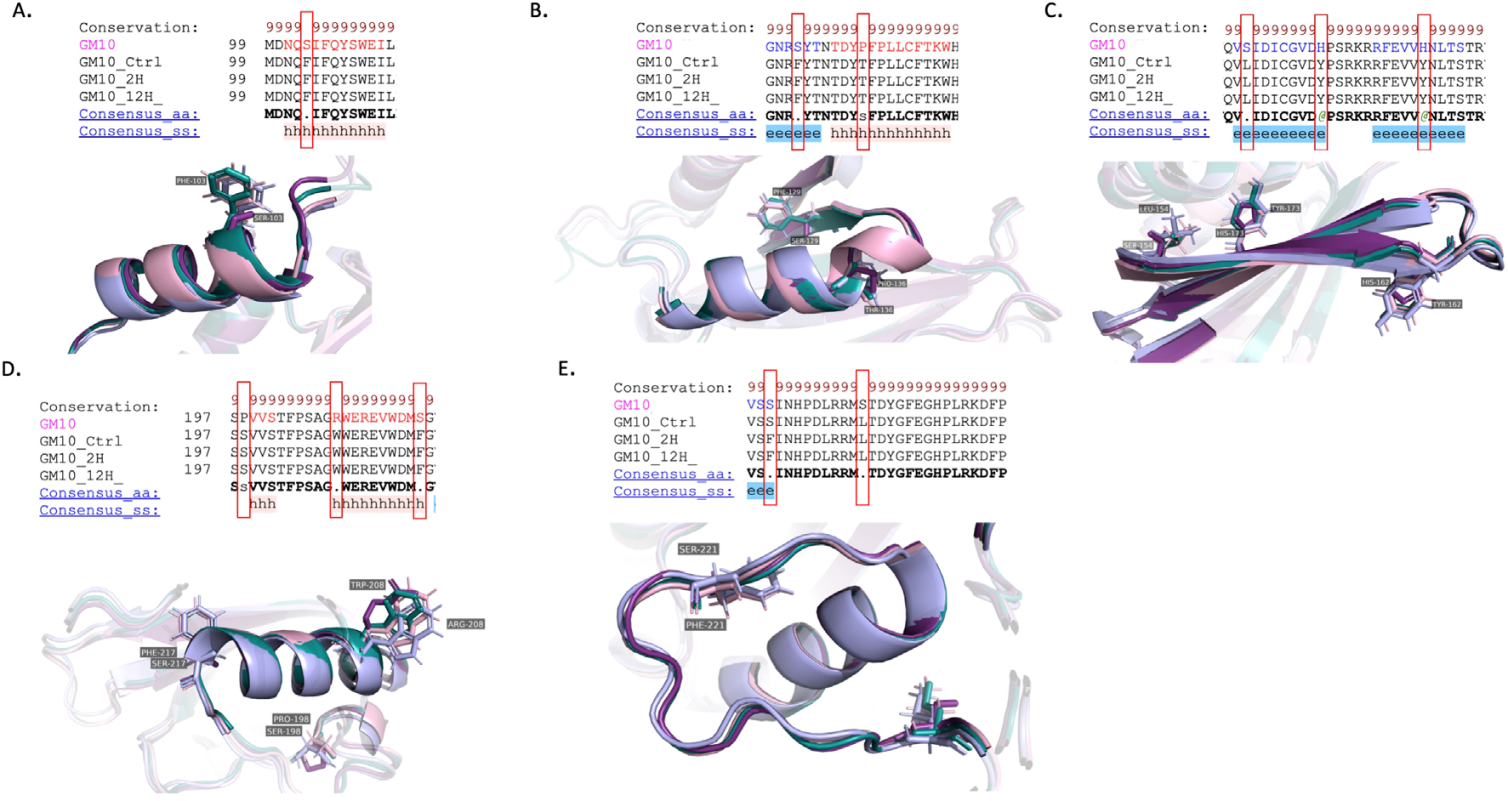
Structural analysis of RNA editing variants in the NAD9 protein of the *Triticum aestivum* Gemmiza10 cultivar across the three drought treatment time points (control, 2 hours, 12 hours). **(A–E)** The panels display a structural alignment of the NAD9 protein virtual translated from DNA (GM-DNA) in comparison with highlighting RNA editing-induced changes in samples collected from the control (GM-C), 2-hour (GM-2H) and 12-hour (GM-12H) post drought treatments. **(A)** Structural comparison of an alpha-helix region across the three time points, illustrating the substitution of an amino acid from the control sequence and its conservation across the treated samples. **(B)** Visualization of another alpha-helical segment where RNA editing modifies residues. **(C)** Beta-strands featuring multiple editing sites, indicating extensive structural alterations post-RNA editing. **(D)** A conserved alpha-helix with RNA editing sites. **(E)** C-terminal beta-strand region, with mapped RNA editing sites.

The Gemmiza10 cultivar harbors a series of RNA editing-induced amino acid modifications at distinct positions that align with critical functional domains of the protein. At position 103, an edit converts a serine (S) to phenylalanine (F), introducing a more hydrophobic residue into the alpha-helical region (**Figure 10A**). Similarly, at position 129, another serine is substituted with phenylalanine, reinforcing the propensity for increased hydrophobic interactions within the protein structure. Moreover, at position 136, a proline (P)-to-threonine (T) transition occurs, which may introduce a new site for potential phosphorylation (**Figure 10B**).

The leucine (L) to serine (S) conversion at position 154 and the histidine (H) to tyrosine (Y) substitution at position 162 could result in significant alterations to the local structural dynamics due to changes in the side chain size (**Figure 10C**). Due to its phenolic group, tyrosine, which is bulky and more reactive, could play a role in enzyme catalysis or in stabilizing protein structure through additional hydrogen bonding.

## 4 Discussion

Identifying RNA editing sites provides novel insights into regulating the *Triticum aestivum nad9* gene, with potential implications for understanding plant responses to stress. RNA editing is crucial for ensuring the correct expression of functional proteins, particularly within mitochondria and chloroplasts. Prior research has established a link between abiotic stress and RNA editing, underscoring the importance of this mechanism in maintaining cellular homeostasis (Yuan and Liu, 2012). NADH dehydrogenase 9 (NAD9) is a mitochondrially encoded subunit of plant respiratory chain complex I (Maldonado et al., 2022). The relationship between RNA editing and plant responses to environmental stress has been extensively studied (Rodrigues et al., 2017; Xiong et al., 2017), highlighting a potential role in stress regulation (Yuan and Liu, 2012). Partial editing of mitochondrial *nad9* transcripts, which encode the NADH dehydrogenase subunit 9, has been observed in several plant species, including potatoes (Grohmann et al., 1994). In this study, we examined RNA editing of the *nad9* gene in response to drought stress in two wheat cultivars, one sensitive and the other tolerant. In the drought-tolerant cultivar Giza168, 22 RNA editing sites were identified, predominantly involving C-to-T transitions (**Figure 1**; **Supplementary Tables S2**, **S4**), consistent with common plant editing mechanisms (Mohammed et al., 2022). Although other types of editing have been reported (Ramadan et al., 2023), the mechanisms underlying these processes remain largely unknown.

Drought stress significantly affected 14 of the 22 editing sites (C178, C208, C308, C386, C517, C553, C592, C604, C673, G807, C820, T837, T855, and C861); however, seven of these sites resulted in synonymous amino acid changes, suggesting an unexplored involvement of tRNA in these processes. A similar pattern was observed in the sensitive cultivar Gemmiza 10, where seven of the 19 identified sites were significantly affected by drought stress (C178, C592, C622, C662, C820, T855, and C861), with synonymous amino acids detected at three sites (C178, T855, and C861). Three critical editing sites distinguished the tolerant cultivar Giza168 from the sensitive cultivar Gemmiza10: C553, where editing leads to a synonymous amino acid change (arginine), but is absent in the sensitive cultivar; C673, which is fully edited in the tolerant cultivar but not in the sensitive one; and C820, where editing increases under stress in the tolerant plants but decreases in the sensitive ones. These findings suggest that drought stress may negatively impact several factors, such as the PPR protein responsible for editing these sites, which may be absent or dysfunctional in sensitive plants (Laluk et al., 2011; Lu et al., 2022; Sun et al., 2018).

Generally, RNA editing directly impacts proteins’ primary, secondary, and tertiary structures. At the primary structure level, amino acids such as arginine 185 (R) to glycine (G), threonine 202 (T) to leucine (L), proline 225 (P) to serine (S), and arginine 274 (R) to cysteine (C) are altered due to drought stress. In the sensitive cultivar, changes such as threonine-60 (T) to leucine (L), serine-221 (S) to phenylalanine (F), and aspartic acid-256 (D) to tyrosine (Y) were observed (**Supplementary Figures S1**, **S2**). Our result, like most RNA editing sites in the mitochondrial *nad* and *atp* genes, resulted in the production of hydrophobic amino acids, which may enhance the affinity of the resulting subunits for the mitochondrial inner membrane (Ibrahim et al., 2023; Rusman et al., 2023).

Drought stress also induces changes in secondary structures, particularly affecting the conformation of alpha helices and beta sheets by altering the amino acid composition. Overall, the protein profiles (beta sheets and alpha helix) of both cultivars were highly similar under control conditions. However, significant differences emerged under stress, both at the 2-hour and 12-hour time points (**Supplementary Figures S5**-**S7**). In the drought-tolerant cultivar Giza168, beta-sheet formation was modified, with one segment becoming two due to an R185G substitution, an increase in beta-sheet length due to a T202L substitution, and the disappearance of alpha helices due to an R274C substitution, however, the P225S substitution preserved the protein loop structure (**Supplementary Figure S3**). In contrast, the sensitive cultivar Gemmiza10 did not exhibit a specific pattern in response to editing, except for an increase in length in some protein regions, such as regions in amino acids 39-94, 212-214, etc. Our study suggests that RNA editing plays a significant role in enhancing plant resilience to drought. If RNA editing fails to improve protein efficiency, the plant may become more susceptible to abiotic stress. These conclusions align with other studies exploring the relationship between RNA editing and environmental stress adaptation (Qin et al., 2021; Ramadan et al., 2023; Xiong et al., 2017).

The predicted secondary structure of the 98 amino acids from multiple sequence alignment underscores the variability and conjectured secondary structure of the 98 N-terminal amino acids, indicating structural plasticity that may facilitate regulatory protein interactions and posttranslational modifications. This level of regulatory sophistication, inferred from sequence variability, indicates the evolutionary adaptations of *Triticum aestivum*, which could profoundly affect the organism’s metabolic efficiency, stress adaptability, and agricultural yield. These insights augment our understanding of the regulatory intricacies governing plant mitochondrial proteins and highlight the potential of these posttranslational modifications as targets for enhancing crop resilience in an era of climatic uncertainty. The elucidation of the structure of plant mitochondrial complex I lagged that of chloroplasts until the complete complex I structure was recently resolved in *Arabidopsis thaliana* via cryo-electron microscopy (cryo-EM) (Klusch et al., 2021). The observed variability in the initial 98 amino acids among the different plant species suggested that the regulatory function of these genes’ merits further investigation. The absence of the first 98 amino acids in the *Arabidopsis* structure, present in *Triticum aestivum* Gemmiza10 and Giza168, presents a unique opportunity to explore species-specific adaptations.

An examination of the N-terminal region of the NAD9 protein in *Triticum aestivum* revealed a complex regulatory environment characterized by posttranslational modifications. Identified phosphorylation sites for GSK3 kinase suggest that phosphorylation events could significantly influence the functions of NAD9. Concurrently, SPOP-binding motifs hint at targeted protein degradation pathways that may be crucial for controlling protein turnover. The ubiquitination mechanism, often a precursor to proteasomal degradation, is hypothesized to play a critical role in regulating mitochondrial function, particularly the specific responses to environmental stress observed in *Triticum aestivum*. These regulatory mechanisms underscore the versatility of the NAD9 protein and its potential importance in plants’ metabolic regulation and ecological response. The coexistence of these regulatory motifs within the NAD9 sequence exemplifies the complexity of cellular control systems, particularly in organisms where metabolic adaptability is essential. The GSK3 (MOD_GSK3_1) motif suggests a modulatory effect on the activity of the NAD9 protein, indicating its involvement in diverse signaling pathways that govern mitochondrial metabolic processes. Previous studies have shown that GSK3 regulates various cellular functions, including metabolism and energy production (Cohen and Frame, 2001; Jope and Johnson, 2004). Given the pivotal function of complex I in cellular energy production (Hirst, 2013), these phosphorylation sites are posited to be critical for the delicate modulation of metabolic activity, which is essential for wheat growth and development. The phosphorylation of mitochondrial proteins by GSK3 has been implicated in regulating ATP production and maintaining mitochondrial integrity (Rusman et al., 2023), further supporting the significance of these sites in the NAD9 protein.

The RNA editing-induced NAD9 protein structural changes of the Giza168 cultivar could significantly impact the protein’s stability by introducing a sulfur-containing side chain (**Figure 9**). These RNA editing events collectively suggest a posttranscriptional modification pattern that may play a critical role in the structural and functional adaptation of the NAD9 protein in response to temporal cues in *Triticum aestivum*. Replacing amino acids within the protein sequence can alter the protein’s physicochemical properties, potentially affecting complex I’s assembly and function in the mitochondria. The S103F amino acid change in Gemmiza10, potentially alters its interaction with the mitochondrial membrane or affect the protein’s stability. Similarly, P136 T may influence the protein’s regulation and interaction with other mitochondrial components (**Figure 10**). This editing event could be critical for modulating the enzyme’s activity in response to cellular energy needs.

Our findings extend the understanding of plant mitochondrial dynamics, highlighting how posttranslational modifications orchestrate energy production and contribute to a plant’s ability to withstand fluctuating conditions. Such insights promise to advance wheat crop resilience, offering a foundation for future research to optimize plant performance in response to global agricultural challenges.

## 5 Conclusion

This study examined RNA editing in the NAD9 in response to drought stress in wheat cultivars, focusing on tolerant and sensitive varieties. The study identified 22 RNA editing sites in the drought-tolerant cultivar Giza168, with 14 sites influenced by drought stress and seven exhibiting synonymous amino acids. Additionally, three critical editing sites were found to differentiate the tolerant cultivar Giza168 from the sensitive cultivar Gemmiza10. Drought stress altered the primary and secondary structures of *nad9* due to changes in alpha helices and beta sheets, with the tolerant cultivar Giza168 experiencing alterations in beta-sheet length and alpha-helix number. In contrast, the sensitive cultivar Gemmiza10 did not exhibit a specific pattern in response to editing, except for an increase in length in some regions. These findings suggest that RNA editing plays a crucial role in enhancing plant drought tolerance, and failure to improve protein efficiency through RNA editing may increase plant susceptibility to abiotic stress.

## Data Availability

The original contributions presented in the study are publicly available. This data can be found at the National Center for Biotechnology Information (NCBI) using accession numbers SRR3089142 - SRR3089153, OQ079951, OQ079955.

## References

[B1] Alexander N. Woetzel N. Meiler J. (2011). “Bcl::Cluster: A method for clustering biological molecules coupled with visualization in the Pymol Molecular Graphics System,” in Proceedings of the 2011 IEEE 1st International Conference on Computational Advances in Bio and Medical Sciences (ICCABS). (Orlando, FL, USA), 13–18. doi: 10.1109/ICCABS.2011.5729867 PMC5091839 27818847

[B2] Chen V. B. Arendall W. B. Headd J. J. Keedy D. A. Immormino R. M. Kapral G. J.. (2010). MolProbity: all-atom structure validation for macromolecular crystallography. Acta Crystallographica Section D: Biol. Crystallogr. 66, 12–21. doi: 10.1107/S0907444909042073 PMC2803126 20057044

[B3] Cohen P. Frame S. (2001). The renaissance of GSK3. Nat. Rev. Mol. Cell Biol. 2, 769–776. doi: 10.1038/35096075 11584304

[B4] Edera A. A. Gandini C. L. Sanchez-Puerta M. V. (2018). Towards a comprehensive picture of C-to-U RNA editing sites in angiosperm mitochondria. Plant Mol. Biol. 97, 215–231. doi: 10.1007/s11103-018-0734-9 29761268

[B5] Ghifari A. S. Murcha M. (2020). Plant mitochondria. eLS. 1 (3), 581–591. doi: 10.1002/9780470015902.a0029217

[B6] Gould C. M. Diella F. Via A. Puntervoll P. Gemünd C. Chabanis-Davidson S.. (2010). ELM: the status of the 2010 eukaryotic linear motif resource. Nucleic Acids Res. 38, D167–D180. doi: 10.1093/nar/gkp1016 19920119 PMC2808914

[B7] Grohmann L. Thieck O. Herz U. Schrüoder W. Brennicke A. (1994). Translation of nad9 mRNAs in mitochondria from Solanum tuberosum is restricted to completely edited transcripts. Nucleic Acids Res. 22, 3304–3311. doi: 10.1093/nar/22.16.3304 8078764 PMC523722

[B8] Hirst J. (2013). Mitochondrial complex I. Annu. Rev. Biochem. 82, 551–575. doi: 10.1146/annurev-biochem-070511-103700 23527692

[B9] Hisano H. Tsujimura M. Yoshida H. Terachi T. Sato K. (2016). Mitochondrial genome sequences from wild and cultivated barley (Hordeum vulgare). BMC Genomics. 17 (1), 824. 27776481 10.1186/s12864-016-3159-3 PMC5078923

[B10] Ibrahim M. I. Ramadan A. M. Amer M. Khan T. K. Mohamed N. G. Said O. A. (2023). Deciphering the enigma of RNA editing in the ATP1_alpha subunit of ATP synthase in Triticum aestivum. Saudi J. Biol. Sci. 30, 103703. doi: 10.1016/j.sjbs.2023.103703 37389198 PMC10300253

[B11] Jope R. S. Johnson G. V. (2004). The glamour and gloom of glycogen synthase kinase-3. Trends Biochem. Sci. 29, 95–102. doi: 10.1016/j.tibs.2003.12.004 15102436

[B12] Jumper J. Evans R. Pritzel A. Green T. Figurnov M. Ronneberger O.. (2021). Highly accurate protein structure prediction with AlphaFold. nature 596, 583–589. doi: 10.1038/s41586-021-03819-2 34265844 PMC8371605

[B13] Klusch N. Senkler J. Yildiz Ö. Kühlbrandt W. Braun H.-P. (2021). A ferredoxin bridge connects the two arms of plant mitochondrial complex I. Plant Cell 33, 2072–2091. doi: 10.1093/plcell/koab092 33768254 PMC8290278

[B14] Laluk K. Abuqamar S. Mengiste T. (2011). The Arabidopsis mitochondria-localized pentatricopeptide repeat protein PGN functions in defense against necrotrophic fungi and abiotic stress tolerance. Plant Physiol. 156, 2053–2068. doi: 10.1104/pp.111.177501 21653783 PMC3149943

[B15] Lu K. Li C. Guan J. Liang W. H. Chen T. Zhao Q. Y.. (2022). The PPR-domain protein SOAR1 regulates salt tolerance in rice. Rice (N Y) 15, 62. doi: 10.1186/s12284-022-00608-x 36463341 PMC9719575

[B16] Maldonado M. Abe K. M. Letts J. A. (2022). A structural perspective on the RNA editing of plant respiratory complexes. Int. J. Mol. Sci. 23, 684. doi: 10.3390/ijms23020684 35054870 PMC8775464

[B17] Mohammed T. Firoz A. Ramadan A. M. (2022). RNA editing in chloroplast: advancements and opportunities. Curr. Issues Mol. Biol. 44, 5593–5604. doi: 10.3390/cimb44110379 36421663 PMC9688838

[B18] Munns R. Millar A. H. (2023). Seven plant capacities to adapt to abiotic stress. J. Exp. Bot. 74, 4308–4323. doi: 10.1093/jxb/erad179 37220077 PMC10433935

[B19] Pandey P. Irulappan V. Bagavathiannan M. V. Senthil-Kumar M. (2017). Impact of combined abiotic and biotic stresses on plant growth and avenues for crop improvement by exploiting physio-morphological traits. Front. Plant Sci. 8. doi: 10.3389/fpls.2017.00537 PMC5394115 28458674

[B20] Pei J. Kim B.-H. Grishin N. V. (2008). PROMALS3D: a tool for multiple protein sequence and structure alignments. Nucleic Acids Res. 36, 2295–2300. doi: 10.1093/nar/gkn072 18287115 PMC2367709

[B21] Qin T. Zhao P. Sun J. Zhao Y. Zhang Y. Yang Q.. (2021). Research progress of PPR proteins in RNA editing, stress response, plant growth and development. Front. Genet. 12, 765580. doi: 10.3389/fgene.2021.765580 34733319 PMC8559896

[B22] Ramadan A. M. (2020). Salinity effects on nad3 gene RNA editing of wild barley mitochondria. Mol. Biol. Rep. 47, 3857–3865. doi: 10.1007/s11033-020-05475-7 32358688

[B23] Ramadan A. M. Mohammed T. Firoz A. Alameldin H. F. Ali H. M. (2023). RNA editing in chloroplast NADH dehydrogenase (ndhA) of salt stressed wild barley revealed novel type G to A. J. King Saud University-Science 35, 102755. doi: 10.1016/j.jksus.2023.102755

[B24] Rasool F. Ishtiaq I. Uzair M. Naz A. A. Léon J. Khan M. R. (2022). Genome-wide investigation and functional analysis of RNA editing sites in wheat. PloS One 17, e0265270. doi: 10.1371/journal.pone.0265270 35275970 PMC8916659

[B25] Rodrigues N. F. Christoff A. P. Da Fonseca G. C. Kulcheski F. R. Margis R. (2017). Unveiling chloroplast RNA editing events using next generation small RNA sequencing data. Front. Plant Sci. 8, 1686. doi: 10.3389/fpls.2017.01686 29033962 PMC5626879

[B26] Rusman F. Floridia-Yapur N. Díaz A. G. Ponce T. Diosque P. Tomasini N. (2023). Hydrophobicity-driven increases in editing in mitochondrial mRNAs during the evolution of kinetoplastids. Mol. Biol. Evol. 40, msad081. doi: 10.1093/molbev/msad081 37030003 PMC10118304

[B27] Slugina M. A. Shchennikova A. V. Kochieva E. Z. (2019). The expression pattern of the Pho1a genes encoding plastidic starch phosphorylase correlates with the degradation of starch during fruit ripening in green-fruited and red-fruited tomato species. Funct. Plant Biol. 46 (12), 1146–1157. doi: 10.1071/FP18317 31615619

[B28] Sun Y. Huang J. Zhong S. Gu H. He S. Qu L. J. (2018). Novel DYW-type pentatricopeptide repeat (PPR) protein BLX controls mitochondrial RNA editing and splicing essential for early seed development of Arabidopsis. J. Genet. Genomics 45, 155–168. doi: 10.1016/j.jgg.2018.01.006 29580769

[B29] The UniProt Consortium. (2023). UniProt: the universal protein knowledgebase in 2023. Nucleic Acids Res. 51, D523–D531. doi: 10.1093/nar/gkac1052 36408920 PMC9825514

[B30] Trenberth K. E. Dai A. van der Schrier G. Jones P. D. Barichivich J. Briffa K. R.. (2014). Global warming and changes in drought. Nat. Climate Change 4, 17–22. doi: 10.1038/nclimate2067

[B31] Tukey J. W. (1949). Comparing individual means in the analysis of variance. Biometrics. 5 (2), 99–114. doi: 10.2307/3001913 18151955

[B32] Wang M. Cui L. Feng K. Deng P. Du X. Wan F.. (2015). Comparative analysis of Asteraceae chloroplast genomes: structural organization, RNA editing and evolution. Plant Mol. Biol. Rep. 33, 1526–1538. doi: 10.1007/s11105-015-0853-2

[B33] Xiong J. Tao T. Luo Z. Yan S. Liu Y. Yu X.. (2017). RNA editing responses to oxidative stress between a wild abortive type male-sterile line and its maintainer line. Front. Plant Sci. 8, 2023. doi: 10.3389/fpls.2017.02023 29234339 PMC5712406

[B34] Yuan H. Liu D. (2012). Functional disruption of the pentatricopeptide protein SLG1 affects mitochondrial RNA editing, plant development, and responses to abiotic stresses in Arabidopsis. Plant J. 70, 432–444. doi: 10.1111/j.1365-313X.2011.04883.x 22248025

