# Supplementary material for: RNA editing-induced structural and functional adaptations of NAD9 in Triticum aestivum under drought stress
Source: Front Plant Sci. 2024 Nov 6;15:1490288. doi: 10.3389/fpls.2024.1490288 (PMC11590480; doi:10.3389/fpls.2024.1490288)
Supplement: Supplementary file 1 [file DataSheet1.pdf]

```
                                                    20                    40                    60
                                                     |                     |                     |
nad9G168 DNA new   ATGCTCTGTA TAATACTTTT CCCCGAGCGA TGGTTTAGCG TATTCGGAAT TGTACCCAAG  60
nad9G168 CONTROL   .......... .......... .......... .......... .......... ..........  60
nad9G1682H         .......... .......... .......... .......... .......... ..........  60
nad9G168 12H       .......... .......... .......... .......... .......... ..........  60
                                                    80                   100                   120
                                                     |                     |                     |
nad9G168 DNA new   CCCTCCCATG GGTTCTATAC CCGATTCATA ACTAGAGCAT GCAGCCGATC CTGGATACAT 120
nad9G168 CONTROL   .......... .......... .......... .......... .......... .......... 120
nad9G1682H         .......... .......... .......... .......... .......... .......... 120
nad9G168 12H       .......... .......... .......... .......... .......... .......... 120
                                                   140                   160                   180
                                                     |                     |                     |
nad9G168 DNA new   AACTCTAAAA AGTGTGTGTG CAGTTTTGGA TCTTTATTGG TAGCCAGTCT TTCACTTCTG 180
nad9G168 CONTROL   .......... .......... .......... .......... .......... .......T.. 180
nad9G1682H         .......... .......... .......... .......... .......... .......T.. 180
nad9G168 12H       .......... .......... .......... .......... .......... .......T.. 180
                                                   200                   220                   240
                                                     |                     |                     |
nad9G168 DNA new   CCTCTCCACT CCCATGCCTT TCTTGGTCGG ACCAACCCAA CCGGCGATTT CCGACAAGTC 240
nad9G168 CONTROL   .......... .......... .......T.. .......... .......... .......... 240
nad9G1682H         .......... .......... .......T.. .......... .......... .......... 240
nad9G168 12H       .......... .......... .......T.. .......... .......... .......... 240
                                                   260                   280                   300
                                                     |                     |                     |
nad9G168 DNA new   TTTCTGCTTA GAGCAAGAAG CGGAACCAAA ATAAAGCTTT CTTTATTTTC ATTTATGGAT 300
nad9G168 CONTROL   .......... .......... .......... .......... .......... .......... 300
nad9G1682H         .......... .......... .......... .......... .......... .......... 300
nad9G168 12H       .......... .......... .......... .......... .......... .......... 300
                                                   320                   340                   360
                                                     |                     |                     |
nad9G168 DNA new   AACCAATCCA TTTTCCAATA TAGTTGGGAG ATTTTACCCA AGAAATGGGT ACATAAAATG 360
nad9G168 CONTROL   .......T.. .......... .......... .......... .......... .......... 360
nad9G1682H         .......T.. .......... .......... .......... .......... .......... 360
nad9G168 12H       .......T.. .......... .......... .......... .......... .......... 360
                                                   380                   400                   420
                                                     |                     |                     |
nad9G168 DNA new   AAAAGATCGG AACATGGGAA TAGATCTTAT ACCAATACTG ACTACCCATT TCCATTGTTG 420
nad9G168 CONTROL   .......... .......... .....T.... .......... ....T.T... .......... 420
nad9G1682H         .......... .......... .....T.... .......... ....T.T... .......... 420
nad9G168 12H       .......... .......... .....T.... .......... ....T.T... .......... 420
                                                   440                   460                   480
                                                     |                     |                     |
nad9G168 DNA new   TGCTTTCTAA AATGGCATAC CTATACAAGG GTTCAAGTTT CGATCGATAT TTGCGGAGTG 480
nad9G168 CONTROL   .......... .......... .......... .......... T......... .......... 480
nad9G1682H         .......... .......... .......... .......... T......... .......... 480
nad9G168 12H       .......... .......... .......... .......... T......... .......... 480
                                                   500                   520                   540
                                                     |                     |                     |
nad9G168 DNA new   GATCATCCCT CTCGAAAACG AAGATTTGAA GTTGTCCATA ATTTACTGAG TACTCGGTAT 540
nad9G168 CONTROL   ...T...... .......... .......... ......T... .......... .......... 540
nad9G1682H         ...T...... .......... .......... ......T... .......... .......... 540
nad9G168 12H       ...T...... .......... .......... ......T... .......... .......... 540
                                                   560                   580                   600
                                                     |                     |                     |
nad9G168 DNA new   AACTCACGCA TTCGTGTACA AACAAGTGCA GACGAAGTAA CACGAATATC TCCGGTAGTC 600
nad9G168 CONTROL   .......... .......... .......... .......... .......... .T........ 600
nad9G1682H         .......... ..G....... .......... .......... .......... .T........ 600
nad9G168 12H       .......... ..G....... .......... .......... .......... .T........ 600
                                                   620                   640                   660
                                                     |                     |                     |
nad9G168 DNA new   AGTCTATTTC CATCAGCCGG CCGGTGGGAG CGAGAAGTAT GGGATATGTC TGGTGTTTCT 660
nad9G168 CONTROL   .......... .......... .T........ .......... .........T .......... 660
nad9G1682H         ...T...... .......... .T........ .......... .........T .......... 660
nad9G168 12H       ...T...... .......... .T........ .......... .........T .......... 660
                                                   680                   700                   720
                                                     |                     |                     |
nad9G168 DNA new   TCCATCAATC ATCCGGATTT ACGCCGTATA TCAACAGATT ATGGTTTCGA GGGTCATCCA 720
nad9G168 CONTROL   .T........ .......... .......... .T........ .......... .......... 720
nad9G1682H         .T........ ..T....... .......... .T........ .......... .......... 720
nad9G168 12H       .T........ ..T....... .......... .T........ .......... .......... 720
                                                   740                   760                   780
                                                     |                     |                     |
nad9G168 DNA new   TTACGAAAAG ACTTTCCTCT GAGTGGATAT GTGGAAGTAC GCTATGATGA TCCAGAGAAA 780
nad9G168 CONTROL   .......... .......... .......... .......... .......... .......... 780
nad9G1682H         .......... .......... .......... .......... .......... .......... 780
nad9G168 12H       .......... .......... .......... .......... .......... .......... 780
                                                   800                   820                   840
                                                     |                     |                     |
nad9G168 DNA new   CGTGTGGTTT CTGAACCCAT TGAGATGACC CAAGAATTTC GCTATTTCGA TTTTGCTAGT 840
nad9G168 CONTROL   .......... .......... .......... .......... .......... .......... 840
nad9G1682H         .......... .......... ......A... .........T .......... ......A... 840
nad9G168 12H       .......... .......... ......A... .........T .......... ......A... 840
                                                   860
                                                     |
nad9G168 DNA new   CCTTGGGAAC AGCGTAGCGA CGGATAA 867
nad9G168 CONTROL   .......... .......... ....... 867
nad9G1682H         .......... ....C..... T...... 867
nad9G168 12H       .......... ....C..... T...... 867
```
