# Supplementary material for: RNA editing-induced structural and functional adaptations of NAD9 in Triticum aestivum under drought stress
Source: Front Plant Sci. 2024 Nov 6;15:1490288. doi: 10.3389/fpls.2024.1490288 (PMC11590480; doi:10.3389/fpls.2024.1490288)
Supplement: Supplementary file 2 [file DataSheet2.pdf]

```
                                        20                    40                    60
                                         |                     |                     |
nad9GM10DNA new  ATGCTCTGTA TAATACTTTT CCCCGAGCGA TGGTTTAGCG TATTCGGAAT TGTACCCAAG 60
nad9GM10control  .......... .......... .......... .......... .......... .......... 60
nad9GM10 2H      .......... .......... .......... .......... .......... .......... 60
nad9GM10 12H     .......... .......... .......... .......... .......... .......... 60

                                        80                   100                   120
                                         |                     |                     |
nad9GM10DNA new  CCCTCCCATG GGTTCTATAC CCGATTCATA ACTAGAGCAT GCAGCCGATC CTGGATACAT 120
nad9GM10control  .......... .......... .......... .......... .......... .......... 120
nad9GM10 2H      .......... .......... .......... .......... .......... .......... 120
nad9GM10 12H     .......... .......... .......... .......... .......... .......... 120

                                       140                   160                   180
                                         |                     |                     |
nad9GM10DNA new  AACTCTAAAA AGTGTGTGTG CAGTTTTGGA TCTTTATTGG TAGCCAGTCT TTCACTTCTG 180
nad9GM10control  .......... .......... .......... .......... .......... .......... 180
nad9GM10 2H      .......... .......... .......... .......... .......... .......T.. 180
nad9GM10 12H     .......... .......... .......... .......... .......... .......T.. 180

                                       200                   220                   240
                                         |                     |                     |
nad9GM10DNA new  CCTCTCCACT CCCATGCCTT TCTTGGTCGG ACCAACCCAA CCGGCGATTT CCGACAAGTC 240
nad9GM10control  .......... .......... .......T.. .......... .......... .......... 240
nad9GM10 2H      .......... .......... .......T.. .......... .......... .......... 240
nad9GM10 12H     .......... .......... .......T.. .......... .......... .......... 240

                                       260                   280                   300
                                         |                     |                     |
nad9GM10DNA new  TTTCTGCTTA GAGCAAGAAG CGGAACCAAA ATAAAGCTTT CTTTATTTTC ATTTATGGAT 300
nad9GM10control  .......... .......... .......... .......... .......... .......... 300
nad9GM10 2H      .......... .......... .......... .......... .......... .......... 300
nad9GM10 12H     .......... .......... .......... .......... .......... .......... 300

                                       320                   340                   360
                                         |                     |                     |
nad9GM10DNA new  AACCAATCCA TTTTCCAATA TAGTTGGGAG ATTTTACCCA AGAAATGGGT ACATAAAATG 360
nad9GM10control  .......T.. .......... .......... .......... .......... .......... 360
nad9GM10 2H      .......T.. .......... .......... .......... .......... .......... 360
nad9GM10 12H     .......T.. .......... .......... .......... .......... .......... 360

                                       380                   400                   420
                                         |                     |                     |
nad9GM10DNA new  AAAAGATCGG AACATGGGAA TAGATCTTAT ACCAATACTG ACTACCCATT TCCATTGTTG 420
nad9GM10control  .......... .......... .....T.... .......... ....T.T... .......... 420
nad9GM10 2H      .......... .......... .....T.... .......... ....T.T... .......... 420
nad9GM10 12H     .......... .......... .....T.... .......... ....T.T... .......... 420

                                       440                   460                   480
                                         |                     |                     |
nad9GM10DNA new  TGCTTTCTAA AATGGCATAC CTATACAAGG GTTCAAGTTT CGATCGATAT TTGCGGAGTG 480
nad9GM10control  .......... .......... .......... .......... T......... .......... 480
nad9GM10 2H      .......... .......... .......... .......... T......... .......... 480
nad9GM10 12H     .......... .......... .......... .......... T......... .......... 480

                                       500                   520                   540
                                         |                     |                     |
nad9GM10DNA new  GATCATCCCT CTCGAAAACG AAGATTTGAA GTTGTCCATA ATTTACTGAG TACTCGGTAT 540
nad9GM10control  ...T...... .......... .......... ......T... .......... .......... 540
nad9GM10 2H      ...T...... .......... .......... ......T... .......... .......... 540
nad9GM10 12H     ...T...... .......... .......... ......T... .......... .......... 540

                                       560                   580                   600
                                         |                     |                     |
nad9GM10DNA new  AACTCACGCA TTCGTGTACA AACAAGTGCA GACGAAGTAA CACGAATATC TCCGGTAGTC 600
nad9GM10control  .......... .......... .......... .......... .......... .T........ 600
nad9GM10 2H      .......... .......... .......... .......... .......... .T........ 600
nad9GM10 12H     .......... .......... .......... .......... .......... .T........ 600

                                       620                   640                   660
                                         |                     |                     |
nad9GM10DNA new  AGTCTATTTC CATCAGCCGG CCGGTGGGAG CGAGAAGTAT GGGATATGTC TGGTGTTTCT 660
nad9GM10control  .......... .......... .T........ .......... .........T .......... 660
nad9GM10 2H      .......... .......... .T........ .......... .........T .......... 660
nad9GM10 12H     .......... .......... .T........ .......... .........T .......... 660

                                       680                   700                   720
                                         |                     |                     |
nad9GM10DNA new  TCCATCAATC ATCCGGATTT ACGCCGTATA TCAACAGATT ATGGTTTCGA GGGTCATCCA 720
nad9GM10control  .......... .......... .......... .T........ .......... .......... 720
nad9GM10 2H      .T........ .......... .......... .T........ .......... .......... 720
nad9GM10 12H     .T........ .......... .......... .T........ .......... .......... 720

                                       740                   760                   780
                                         |                     |                     |
nad9GM10DNA new  TTACGAAAAG ACTTTCCTCT GAGTGGATAT GTGGAAGTAC GCTATGATGA TCCAGAGAAA 780
nad9GM10control  .......... .......... .......... .......... .......... .......... 780
nad9GM10 2H      .......... .......... .......... .......... .......... .......... 780
nad9GM10 12H     .......... .......... .......... .......... .....T.... .......... 780

                                       800                   820                   840
                                         |                     |                     |
nad9GM10DNA new  CGTGTGGTTT CTGAACCCAT TGAGATGACC CAAGAATTTC GCTATTTCGA TTTTGCTAGT 840
nad9GM10control  .......... .......... ......A... .........T .......... ......A... 840
nad9GM10 2H      .......... .......... ......A... .......... .......... ......A... 840
nad9GM10 12H     .......... .......... ......A... .......... .......... ......A... 840

                                       860
                                         |
nad9GM10DNA new  CCTTGGGAAC AGCGTAGCGA CGGATAA 867
nad9GM10control  .......... ....C..... T...... 867
nad9GM10 2H      .......... ....C..... T...... 867
nad9GM10 12H     .......... ....C..... T...... 867
```
