# Supplementary figures and images for: RNA editing-induced structural and functional adaptations of NAD9 in Triticum aestivum under drought stress
Source: Front Plant Sci. 2024 Nov 6;15:1490288. doi: 10.3389/fpls.2024.1490288 (PMC11590480; doi:10.3389/fpls.2024.1490288)

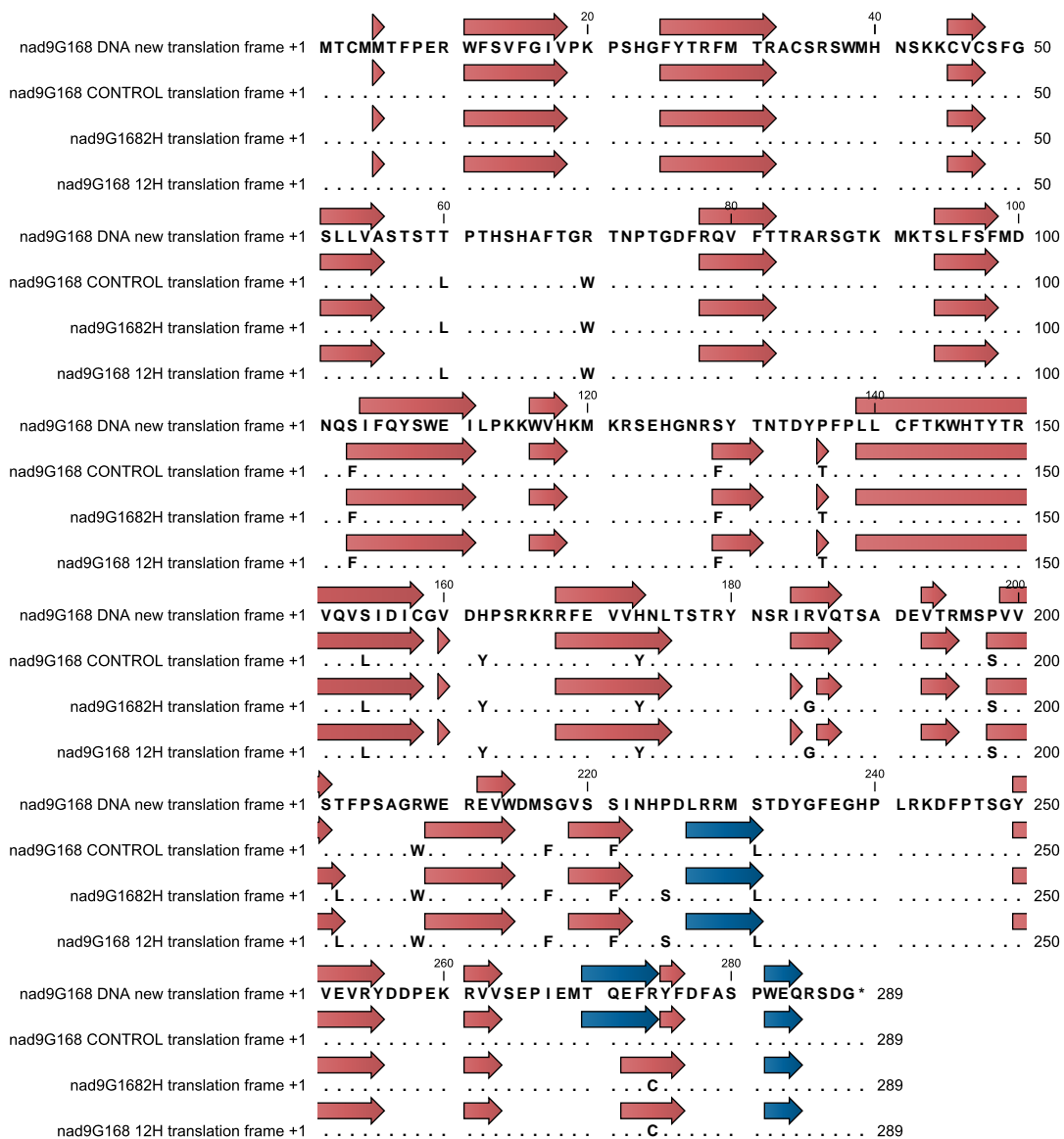

Supplement: Supplementary file 3 [file DataSheet3.pdf]

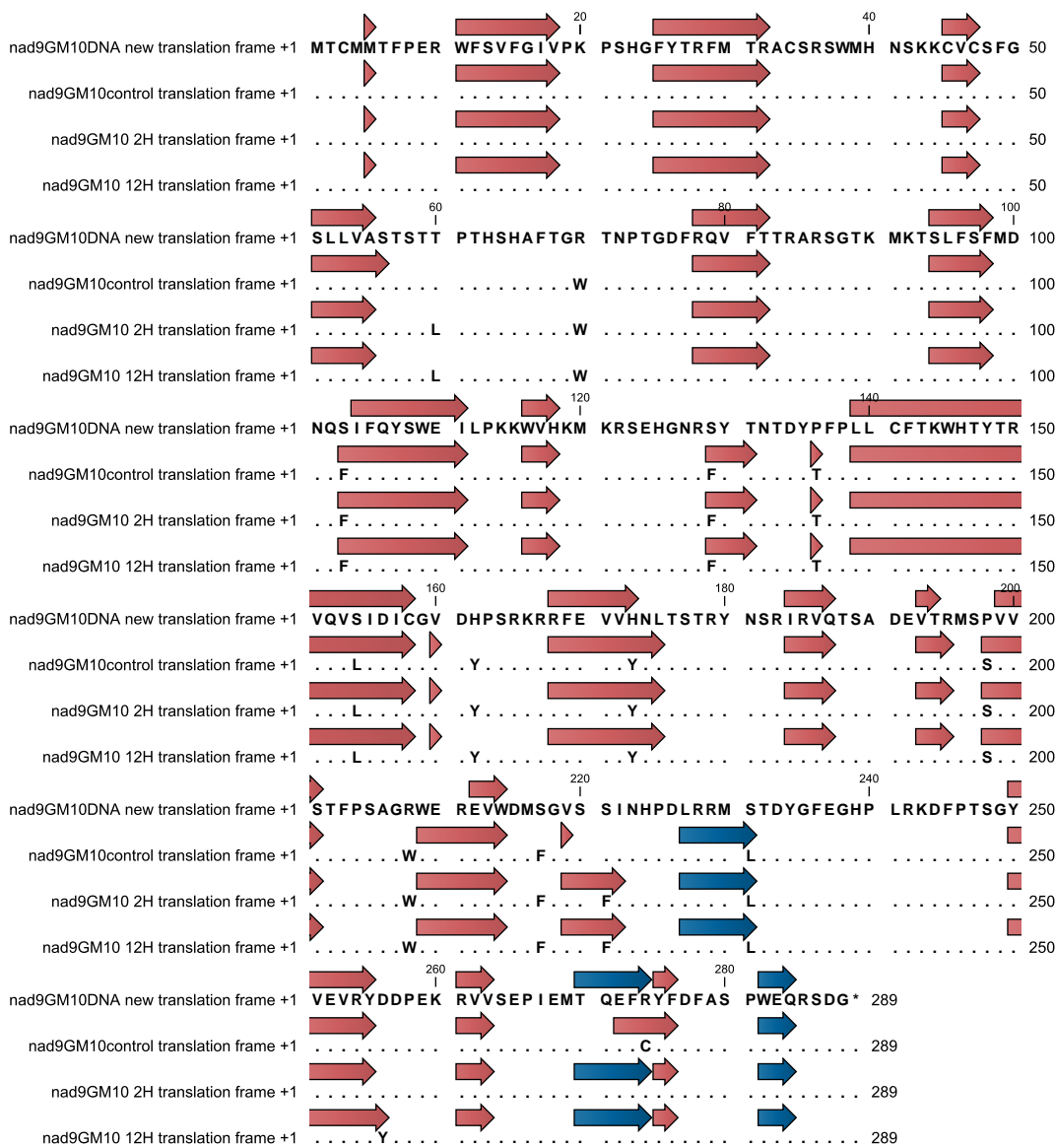

Supplement: Supplementary file 4 [file DataSheet4.pdf]

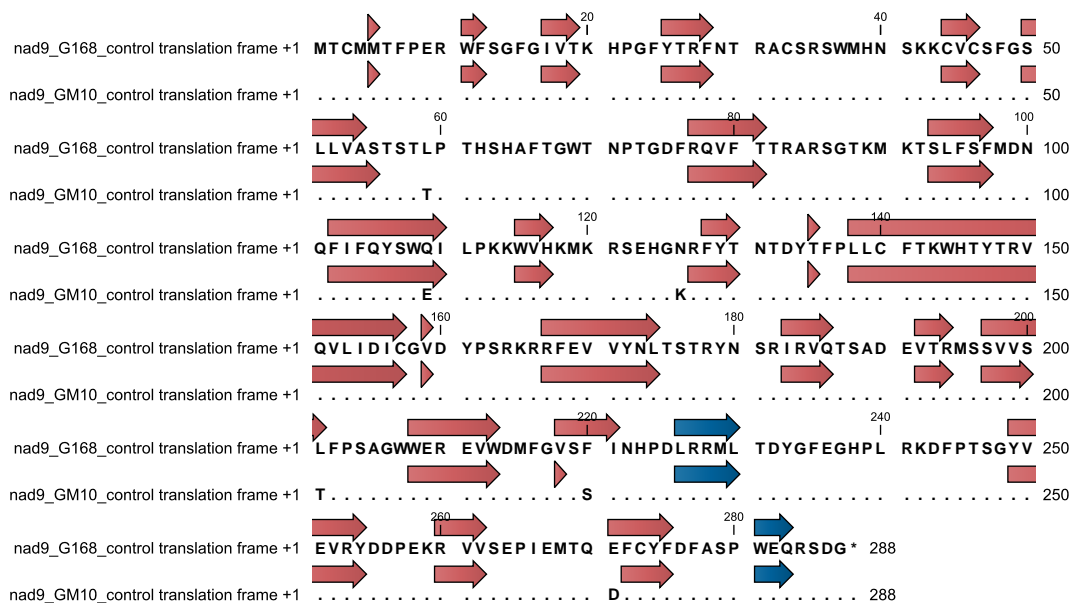

Supplement: Supplementary file 5 [file DataSheet5.pdf]
