# Supplementary material for: RNA editing-induced structural and functional adaptations of NAD9 in Triticum aestivum under drought stress
Source: Front Plant Sci. 2024 Nov 6;15:1490288. doi: 10.3389/fpls.2024.1490288 (PMC11590480; doi:10.3389/fpls.2024.1490288)
Supplement: Supplementary file 7 [file DataSheet7.pdf]

```
nad9_G168_12H translation frame +1  MTCMMTFPER WFSGFGIVTK HPGFYTRFNT RACSRSWMHN SKKCVCSFGS 50
nad9_GM10_12H translation frame +1  .......... .....E.... .......... .......... .......... 50

nad9_G168_12H translation frame +1  LLVASTSTTP THSHAFTGWT NPTGDLRQVF TTRARSGTKM KTSLFSFMDN 100
nad9_GM10_12H translation frame +1  ........L. .......... .....F.... .......... .......... 100

nad9_G168_12H translation frame +1  QFIFQYSWEI LPKKWVHKMK RSEHGNRSYT NTDYPFPLLC FTKWHTYTRV 150
nad9_GM10_12H translation frame +1  .......... .......... .......F.. ....T..... .......... 150

nad9_G168_12H translation frame +1  QVSIDICGVD YPSRKRRFEV VYNLTSTRYN SRIRVQTSAD EVTRMSSVVS 200
nad9_GM10_12H translation frame +1  ..L....... .......... .H........ .......... .......... 200

nad9_G168_12H translation frame +1  TFPSAGWWER EVWDMFGVSF INHPDLRRML TDYGFEGHPL RKDFPTSGYV 250
nad9_GM10_12H translation frame +1  .......... .........S .......... .......... .......... 250

nad9_G168_12H translation frame +1  EVRYYDPEKR VVSEPIEMTQ EFCYFDFASP WEQRSDG* 288
nad9_GM10_12H translation frame +1  .......... .......... ..R....... ........ 288
```
