# Supplementary material for: RNA editing-induced structural and functional adaptations of NAD9 in Triticum aestivum under drought stress
Source: Front Plant Sci. 2024 Nov 6;15:1490288. doi: 10.3389/fpls.2024.1490288 (PMC11590480; doi:10.3389/fpls.2024.1490288)
Supplement: Supplementary file 8 [file Table1.docx]

| pos | F | R |
| --- | --- | --- |
| C178 | TGGTAGCCAGTCTTTCACTTC  TGGTAGCCAGTCTTTCACTT**T** | GGAAAATGGATTGGTTATCCA |
| C208 | TCCCATGCCTTTCTTGGTC  TCCCATGCCTTTCTTGGT**T** | CTTGGGTAAAATCTCCCAA |
| C308 | TCATTTATGGATAACCAATC  TCATTTATGGATAACCAAT**T** | TGTATAGGTATGCCATTTTAG |
| C386 | TCGGAACATGGGAATAGATC  TCGGAACATGGGAATAGAT**T** | GTAAATTATGGACAACTTCA |
| C405 | CTTATACCAATACTGACTAC  CTTATACCAATACTGACTA**T** | GTTATACCGAGTACTCAGT |
| C407 | TATACCAATACTGACTACCC  TATACCAATACTGACTACC**T** | GTTATACCGAGTACTCAGT |
| C461 | TATACAAGGGTTCAAGTTTC  TATACAAGGGTTCAAGTTT**T** | GACTACCGGAGATATTCGT |
| C484 | TCGATATTTGCGGAGTGGATC  TCGATATTTGCGGAGTGGAT**T** | CTGATGGAAATAGACTGACT |
| C517 | ACGAAGATTTGAAGTTGTCC  ACGAAGATTTGAAGTTGTC**T** | ACACCAGACATATCCCATAC |
| C553 | TCGGTATAACTCACGCATTC  TCGGTATAACTCACGCATT**T** | TATACGGCGTAAATCCGGATG |
| C592 | ACGAAGTAACACGAATATCTC  ACGAAGTAACACGAATATCT**T** | CGTAATGGATGACCCTCGA |
| C604 | AATATCTCCGGTAGTCAGTC  AATATCTCCGGTAGTCAGT**T** | GAGGAAAGTCTTTTCGTAA |
| C622 | CTATTTCCATCAGCCGGCC  CTATTTCCATCAGCCGGC**T** | GTACTTCCACATATCCACTCA |
| C650 | GCGAGAAGTATGGGATATGTC  GCGAGAAGTATGGGATATGT**T** | CGTTTCTCTGGATCATCATA |
| C662 | GATATGTCTGGTGTTTCTTC  GATATGTCTGGTGTTTCTT**T** | ATGGGTTCAGAAACCACAC |
| C673 | GTGTTTCTTCCATCAATCATC  GTGTTTCTTCCATCAATCAT**T** | GTCATCTCAATGGGTTCAGA |
| C692 | TCCGGATTTACGCCGTATATC  TCCGGATTTACGCCGTATAT**T** | GAAATAGCGAAATTCTTGGGT |
| G807 | CATCCGGATTTACGCCGTATA | ATAGCGAAATTCTTGGGTC  ATAGCGAAATTCTTGGGT**T** |
| C820 | CCGGATTTACGCCGTATAT | TAGCAAAATCGAAATAGCG  TAGCAAAATCGAAATAGC**A** |
| T837 | ACAGATTATGGTTTCGAGG | ACGCTGTTCCCAAGGACTA  ACGCTGTTCCCAAGGACT**T** |
| T855 |  |  |
| C861 |  |  |
| actin | TGACGTGGATATCAGGAAGG | GCTGAGTGAGGCTAGGATGG |

G168

Gm10

| pos | F | R |
| --- | --- | --- |
| C178 | TGGTAGCCAGTCTTTCACTTC  TGGTAGCCAGTCTTTCACTT**T** | GGAAAATGGATTGGTTATCCA |
| C208 | TCCCATGCCTTTCTTGGTC  TCCCATGCCTTTCTTGGT**T** | CTTGGGTAAAATCTCCCAA |
| C308 | TCATTTATGGATAACCAATC  TCATTTATGGATAACCAAT**T** | TGTATAGGTATGCCATTTTAG |
| C386 | TCGGAACATGGGAATAGATC  TCGGAACATGGGAATAGAT**T** | GTAAATTATGGACAACTTCA |
| C405 | CTTATACCAATACTGACTAC  CTTATACCAATACTGACTA**T** | GTTATACCGAGTACTCAGT |
| C407 | TATACCAATACTGACTACCC  TATACCAATACTGACTACC**T** | GTTATACCGAGTACTCAGT |
| C461 | TATACAAGGGTTCAAGTTTC  TATACAAGGGTTCAAGTTT**T** | GACTACCGGAGATATTCGT |
| C484 | TCGATATTTGCGGAGTGGATC  TCGATATTTGCGGAGTGGAT**T** | CTGATGGAAATAGACTGACT |
| C517 | ACGAAGATTTGAAGTTGTCC  ACGAAGATTTGAAGTTGTC**T** | ACACCAGACATATCCCATAC |
| C592 | ACGAAGTAACACGAATATCTC  ACGAAGTAACACGAATATCT**T** | CGTAATGGATGACCCTCGA |
| C622 | CTATTTCCATCAGCCGGCC  CTATTTCCATCAGCCGGC**T** | GTACTTCCACATATCCACTCA |
| C650 | GCGAGAAGTATGGGATATGTC  GCGAGAAGTATGGGATATGT**T** | CGTTTCTCTGGATCATCATA |
| C662 | GATATGTCTGGTGTTTCTTC  GATATGTCTGGTGTTTCTT**T** | ATGGGTTCAGAAACCACAC |
| C692 | TCCGGATTTACGCCGTATATC  TCCGGATTTACGCCGTATAT**T** | GAAATAGCGAAATTCTTGGGT |
| G807 | CATCCGGATTTACGCCGTATA | ATAGCGAAATTCTTGGGTC  ATAGCGAAATTCTTGGGT**T** |
| C820 | CCGGATTTACGCCGTATAT | TAGCAAAATCGAAATAGCG  TAGCAAAATCGAAATAGC**A** |
| T837 | ACAGATTATGGTTTCGAGG | ACGCTGTTCCCAAGGACTA  ACGCTGTTCCCAAGGACT**T** |
| T855 |  |  |
| C861 |  |  |
| actin | TGACGTGGATATCAGGAAGG | GCTGAGTGAGGCTAGGATGG |
