# Supplementary material for: RNA editing-induced structural and functional adaptations of NAD9 in Triticum aestivum under drought stress
Source: Front Plant Sci. 2024 Nov 6;15:1490288. doi: 10.3389/fpls.2024.1490288 (PMC11590480; doi:10.3389/fpls.2024.1490288)
Supplement: Supplementary file 6 [file DataSheet6.pdf]

```
nad9_G168_2H translation frame +1  MTCMMTFPER WFSGFGIVTK HPGFYTRFNT RACSRSWMHN SKKCVCSFGS  50
nad9_GM10_2H translation frame +1  .......... .......... .......... .......... ..........  50

nad9_G168_2H translation frame +1  LLVASTSTTP THSHAFTGRT NPTGDFRQVF TTRARSGTKM KTSLFSFMDN 100
nad9_GM10_2H translation frame +1  ........L. .Y......W. .......... .......... .......... 100

nad9_G168_2H translation frame +1  QSIFQYSWEI LPKKWVHKMK RSEHGNRSYT NTDYPFPLLC FTKWHTYTRV 150
nad9_GM10_2H translation frame +1  .F........ .......... .......F.. ....T..... .......... 150

nad9_G168_2H translation frame +1  QVSIDICGVD YPSRKRRFEV VYNLTSTRYN SRIGVQTSAD EVTRMSSVVS 200
nad9_GM10_2H translation frame +1  ..L....... .......... .......... ...R...... .......... 200

nad9_G168_2H translation frame +1  LFPSAGWWER EVWDMFGVSF INHSDLRRML TDYGFEGHPL RKDFPTSGYV 250
nad9_GM10_2H translation frame +1  .......... .......... ...P...... .......... .......... 250

nad9_G168_2H translation frame +1  EVRYDDPEKR VVSEPIEMTQ EFCYFDFASP WEQRSDG* 288
nad9_GM10_2H translation frame +1  .......... .......... ..R....... ........ 288
```
